# Isolation and characterization of a novel oligomeric proanthocyanidin with significant anti-cancer activities from grape stems (*Vitis vinifera*)

**DOI:** 10.1038/s41598-019-48603-5

**Published:** 2019-08-19

**Authors:** Kawahara Sei-ichi, Kazuya Toda, Kiriko Matsumoto, Chisato Ishihara, Shuhei Nonobe, Chisato Matsunaga, Yoshika K. Gomi, Shogo Senga, Koichiro Kawaguchi, Ayaka Yamamoto, Jutalak Suwannachot, Reiko Noda, Shuhei Kobayashi, Yasunori Hamauzu, Hidefumi Makabe, Hiroshi Fujii

**Affiliations:** 1 Interdisciplinary Graduate School of Science and Technology, Shinshu University, 8304 Minami-minowa Kami-ina, Nagano, 399-4598 Japan; 2 St. Cousair Co., Ltd., 1260 Imogawa, Kami-minochi, Iizuna, Nagano 389-1201 Japan; 3 Department of Bioscience and Biotechnology, Faculty of Agriculture, Shinshu University, 8304 Minami-minowa Kami-ina, Nagano, 399-4598 Japan; 4 Graduate School of Science and Technology, Department of Biomedical Engineering, Shinshu University, 8304 Minami-minowa Kami-ina, Nagano, 399-4598 Japan; 5 Graduate School of Science and Technology, Department of Agriculture, Division of Food Science and Biotechnology, Shinshu University, 8304 Minami-minowa Kami-ina, Nagano, 399-4598 Japan; 6 Department of Interdisciplinary Genome Sciences and Cell Metabolism, Institute for Biomedical Sciences, Interdisciplinary Cluster for Cutting Edge Research, Shinshu University, Minami-minowa, Kami-ina, Nagano 399-4598 Japan

**Keywords:** Biochemistry, Molecular biology, Cancer prevention

## Abstract

Novel proanthocyanidin fractions from grape stem extracts were purified using Amberlite XAD-1180N, Sephadex-LH-20, Toyopearl HW40F and reverse phase high-performance liquid chromatography. Two key compounds were estimated as epigallocatechin-(epicatechin)_7_ gallate using electron-spray ionization time-of-flight mass spectrometry. Epigallocatechin-(epicatechin)_7_ gallate (compound **1**) showed significant anti-cancer activity in PC-3 prostate cancer cells. In particular, compound **1** suppressed the gene expression of fatty acid-binding protein 5 (*FABP5*), which is involved in promoting cell proliferation and metastasis in prostate cancer cells.

## Introduction

Flavan-3-ols are a group of polymers that are widely distributed as secondary metabolites in plants, including grape stems. Proanthocyanidins are flavan-3-ols consisting of (+)-catechin, (−)-epicatechin, (+)-gallocatechin, and (−)-epigallocatechin units that are linked together through the 4–8 or 4–6 inter-flavan bonds. Proanthocyanidins exist as oligomers and polymers with a wide range of degree of polymerization^1,2^. The structural diversity of these compounds is due to the linkage of inter-flavan bonds and the degree of oligomerization. Proanthocyanidins reportedly show various biological activities such as the suppression of endotherin-1 gene expression^3^, anti-inflammatory effects^4^ and inhibition of cancer cell proliferation^5–10^. We recently reported that epicatechin pentamers and hexamers inhibited the invasive activities of cancer cells^11^. Structural elucidation of proanthocyanidins in natural product mixtures is challenging despites the use of modern analytical techniques. The use of the high-performance liquid chromatography (HPLC) coupled with mass spectrometry apparently provides an efficient and robust approach for structural elucidation.

The aim of this work was to separate, characterize and evaluate the antitumor activity of the isolated compounds from grape stems. Gel filtration chromatography and HPLC was successfully applied in the present study to isolate a novel proanthocyanidin. This compound was octameric epicatechins with an epigallocatechin unit and a gallate unit, respectively. The compound showed significant cytotoxic activity against PC-3 cells. Moreover, the compound suppressed the expression of the cancer-promoting gene *FABP5*^12,13^ and exhibited superior activity compared with that of epigallocatechin 3-gallate (EGCG).

## Results

### Isolation and characterization of a novel oligomeric proanthocyanidin with significant anti-cancer activities from grape stem (*Vitis vinifera*)

The main interest of this research was the characterization and evaluation of the antitumor activity of the proanthocyanidin present in the grape stems. To the best of our knowledge, this is the first report to characterize a proanthocyanidin from grape stems with a structure of epicatechin octamer (compound **1**) that possesses a pyrogallol moiety and a gallate.

The HPLC chromatogram of crude grape stem extracts is shown in Supplementary Fig. 1. The purification scheme is shown in Supplementary Fig. 2. We identified catechin, epicatechin, gallocatechin, EGCG, piceid, and dehydroquercetin rhamnoside. These compounds were analysed using ESI-TOFMS and NMR, and compared with the spectra of known compounds. Spectra of the dimeric to tetrameric units of epicatechin or catechin were obtained from synthesized materials^11,14,15^. Purification using Abmerlite XAD-1180N for the first stage with water followed by AcOEt, and MeOH as eluents afforded three fractions. As shown in Supplementary Fig. 3, low molecular weight compounds such as epicatechin monomers and dimers were included in the AcOEt fraction. A broad peak was observed around 55 min, as shown in Supplementary Fig. 1. This fraction contained higher molecular weight compounds such as highly oligomerised proanthocyanidins. The HPLC chromatogram of this fraction is shown in Supplementary Fig. 4. Remarkably, this fraction showed the significant suppressive activity on the expression of the cancer-promoting genes, *FABP5*. Thus, this fraction was further purified with Sephadex LH 20 using 30% MeOH, 40% EtOH, 30% acetone, 40% acetone, and 60% acetone. The most bioactive fraction was the 60% acetone fraction. This fraction was further purified with Toyopearl HW 40 F using 30% MeOH, 40% EtOH, and 60% acetone. The most bioactive fraction was the 60% acetone fraction. The suppressive activities of each bioactive fraction against *FABP5* are summarized in Supplementary Fig. 5. The HPLC chromatogram of this fraction showed two major peaks (Supplementary Fig. 6). The ESI-TOMS analysis of this fraction is shown in Supplementary Fig. 7. The MS spectrum showed the existence of catechin or epicatechin oligomers with gallocatechin or epigallocatechin and gallate (Supplementary Fig. 7). The bioactive compounds were estimated to be proanthocyanidin oligomers. Thus, we attempted to isolate the compounds using preparative reverse phased HPLC. Although separation of these two peaks was quite difficult, we managed to obtain compound **1** (15 mg) (Supplementary Fig. 8). The ^1^H NMR analysis of compound **1** did not allow for elucidation of the structure because of severe broadening of signals (Supplementary Fig. 9a). We tried to obtain well resolved ^1^H NMR spectra measured at low temperatures, as Witerhalter and co-workers reported that well resolved ^1^H NMR data was obtainable at low temperature^16^. However, even at low temperature (−75 to 0 °C), the broadening of the peaks was not improved in our case. The ^13^C NMR of compound **1** was similar to those of epicatechin oligomers (Supplementary Fig. 9b)^17^. Although the ^13^C NMR spectra of grape stem extracts delivered valuable information about some chemical characteristics, the oligomeric structures occurring in condensed tannin could not be exactly determined exactly due to the broadening signals of ^1^H NMR.

**Figure 1:**
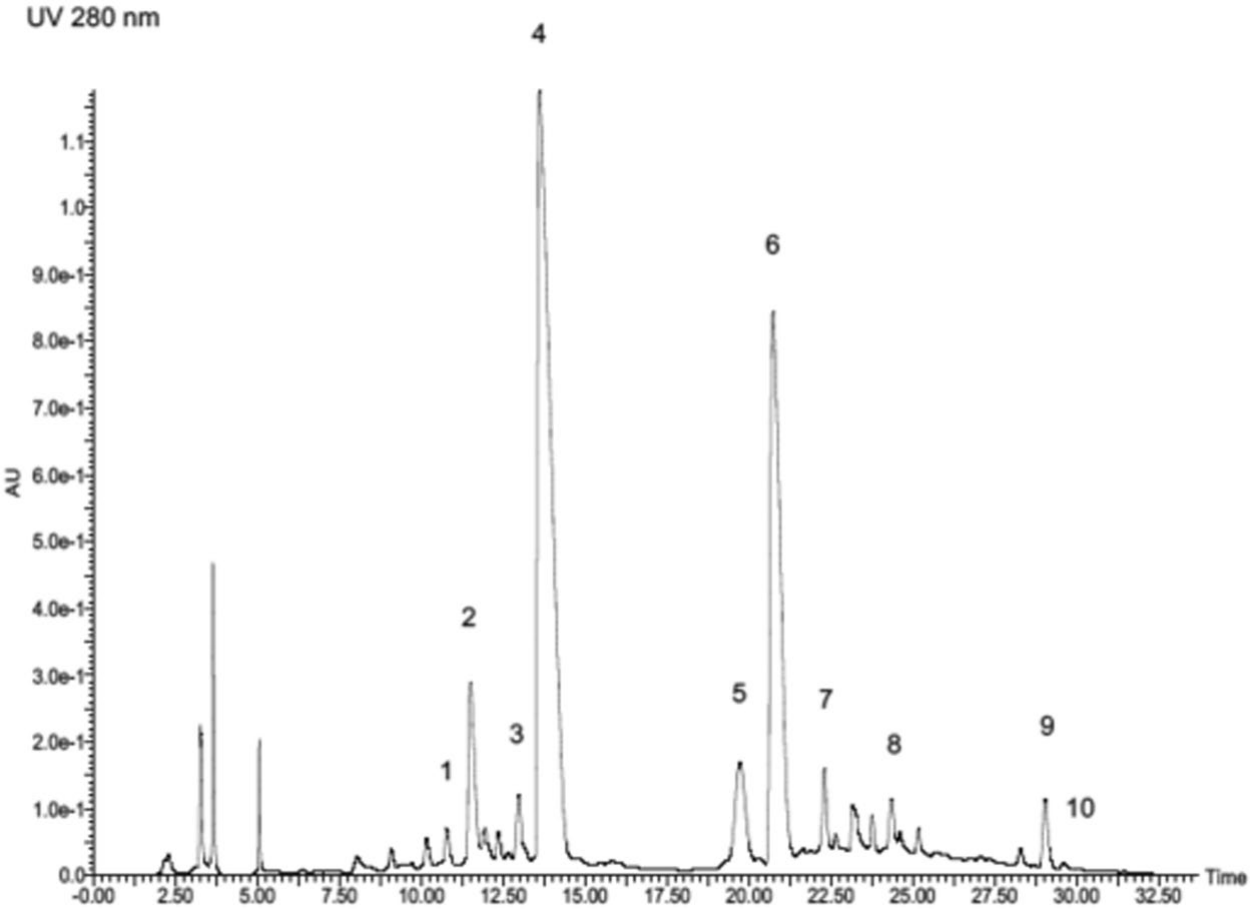
HPLC chromatogram of thiolytic degraded products of compound 1. Peaks: 1 = epigallocatechin-cysteamine thioether, 2 = catechin-cysteamine thioether, 3 = epigallocatechin-gallate-cysteamine thioether, 4 = epicatechin-cysteamine thioether, 5 = catechin, 6 = epicatechin-gallate-cysteamine thioether, 7 = catechin-gallate-cysteamine thioether, 8 = epicatechin, 9 = epicatechin-gallate, 10 = catechin-gallate.

Next, we performed thiolysis of compound **1** to elucidate the structure of extension and terminal unit of grape stem extract. The identification of (−)-epicatechin, (+)-catechin, (−)-epicatechin gallate, and (−)-epigallocatechin and their cysteamine adducts was performed by comparison with the thiolytic products of synthetic epicatechin dimer, catechin dimer, epicatechin-gallate dimer and epigallocatechin dimer (Supplementary Fig. 10f)^10,18,19^. Thiolysis of compound **1** gave (−)-epicatechin-cysteamine thioether as the main degradation product (peak 4 in Fig. 1) whose molecular ion was observed at *m/z* 364 ([M − H]^−^)^20^. This further released a fragment ion at *m/z* 287 in the negative mode. This characteristic fragmentation of (−)-epicatechin-cysteamine thioether is in line with the report by Selga *et al*.^21^ (−)-Epicatechin-gallate-cysteamine thioether (peak 6, [M − H]^−^ at *m/z* 516) was also detected as the extension unit, despite its lower level compared with epicatechin-cysteamine thioether. Moreover, small amount of catechin-cysteamine thioether (peak 2, [M − H]^–^ at *m/z* 364, fragment ion at m/z 287), epigallocatechin-cysteamine thioether (peak 1, [M − H]^−^ at *m/z* 380, fragment ion at *m/z* 303) epigallocatechin-gallate-cysteamine thioether (peak 3, [M − H]^−^ at *m/z* 532), and catechin-gallate-cysteamine thioether (peak 7, [M − H]^−^ at *m/z* 516) were also detected. We also detected (−)-epicatechin-gallate and (+)-catechin-gallate (peak 9 and 10, [M − H]^−^ at *m/z* 441), (+)-catechin and (−)-epicatechin (peak 5 and 8, respectively, [M − H]^−^ at *m/z* 289) as thiolysis products without cysteamine conjugation, indicating that these are candidates for terminal units of grape stem extract proanthocyanidins. While (−)-epicatechin could potentially be epimerized to (+)-catechin during thiolysis at 75 °C, the (+)-catechin peak could not be eliminated in reaction under 50 °C. However, the catechin-gallate peak could be reduced by thiolysis at 50 °C, suggesting that epicatechin-gallate may be the actual unit and the intensity of catechin-gallate could have been artificially increased. Thus, the results suggested that compound **1** consisted of (−)-epicatechin as the major extension unit and (−)-epigallocatechin as the minor extension unit. The terminal units comprised mainly (−)-epicatechin-gallate and (−)-epicatechin and possibly (+)-catechin (Fig. 1, Supplementary Fig. 10a–f). The A-type linkage in proanthocyanidins remains stable during the thiolytic degradation^22,23^. The A-type linkage in terminal units is released as an A-type dimer, whereas the A-type linkage between the extension units yields an A-type dimeric thioether. In our thiolysis result, A-type dimer derivatives were not observed as main peaks. Thus, the isolated major product has B type inter-flavan bonds.

LC-ESI-TOFMS at cationic mode was also performed in the cationic mode to characterize compound **1** (Supplementary Fig. 11). The molecular ion of compound **1** was detected at *m/z* 2475, which fit well with an epigallocatechin-(epicatechin)_7_ gallate. The thiolysis result of compound **1** suggested the main structural unit to be (−)-epicatechin. Its MS/MS spectra gave daughter ion peaks at 2187.4 [M−epicatechin + H], 2171.5 [M − epigallocatechin + H], 1899.4 [M − (epicatechin)_2_ + H], 1883.4 [M − (epicatechin-epigallocatechin) + H], 1747.4 [M − (epicatechin)_2_-gallate + H], 1611.3 [M − (epicatechin)_3_ + H], 1595.3 [M − (epicatechin)_2_-epigallocatechin + H], 1443.3 [M − (epicatechin)_2_-epigallocatechin-gallate + H], 1323.3 [M − (epicatechin)_4_ + H], 1307.3 [M − (epicatechin)_3_-epigallocatechin + H], 1019.2 [M − (epicatechin)_4_-epigallocatechin + H], 867 [M − (epicatechin)_4_-epigallocatechin-gallate + H], 579 [M − (epicatechin)_5_-epigallocatechin-gallate + H] (Supplementary Fig. 12). HRESI-TOFMS: Calcd for C_127_H_102_O_54_ [M + H]^+^, 2475.5364; found, 2475.5425. UVλ_max_nm: 280; IR (KBr)*v*_max_ cm^−1^: 3360, 2981, 2925, 1692, 1613, 1433, 1370, 1228, 1038; the optical rotation value was +121 (*c* 0.15, MeOH)^6^. Therefore, compound **1** was estimated to be epigallocatechin-(epicatechin)_7_ gallate. The position of epigallocatechin and the linking of 4–8 or 4–6 inter-flavan bonds could not be determined due to the broad peaks of ^1^H NMR.

### Compound 1 suppresses cell proliferation of PC-3 cells

**Figure 2:**
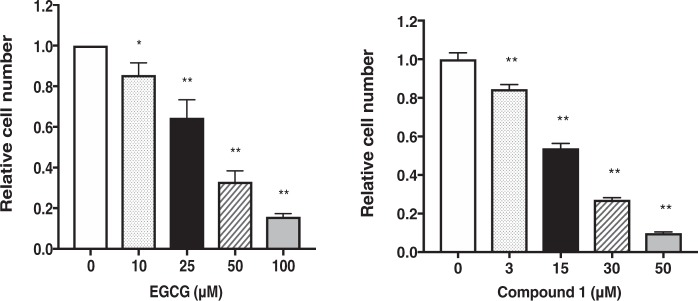
Effects of various concentrations of EGCG and compound 1 on PC-3 cell proliferation. After treatment of cells with either EGCG or compound **1** for 48 h, cell proliferation was evaluated by cell count. The values are presented as the rate of inhibition of cell proliferation in the treated samples compared with that in the control (vehicle). Values are expressed as means ± S.D. of three independent experiments. One-way ANOVA followed by Dunnett’s multiple comparison test was used for multiple comparisons. *P < 0.05, **P < 0.01.

**Figure 3:**
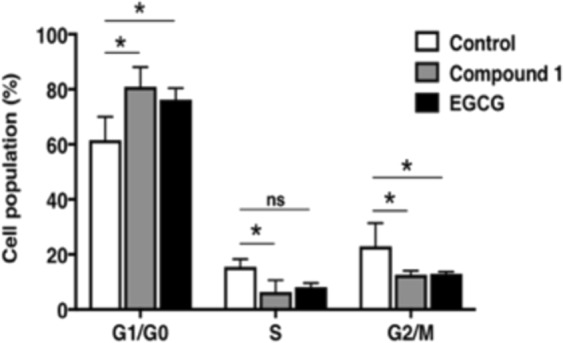
Effects of EGCG and compound 1 on cell cycle distribution. Cells treated with 30 μmol/L of EGCG or compound **1** for 48 h were stained with propidium iodide using a BD Cycletest Plus DNA Reagent Kit (Becton Dickinson and Company BD Biosciences) obtained from Phoenix Flow Systems. Following FACS analysis, cell cycle distributions were further analysed by Cell Quest software. *P < 0.05, two-way ANOVA followed by Tukey’s multiple comparison test. Ns, not significant.

**Figure 4:**
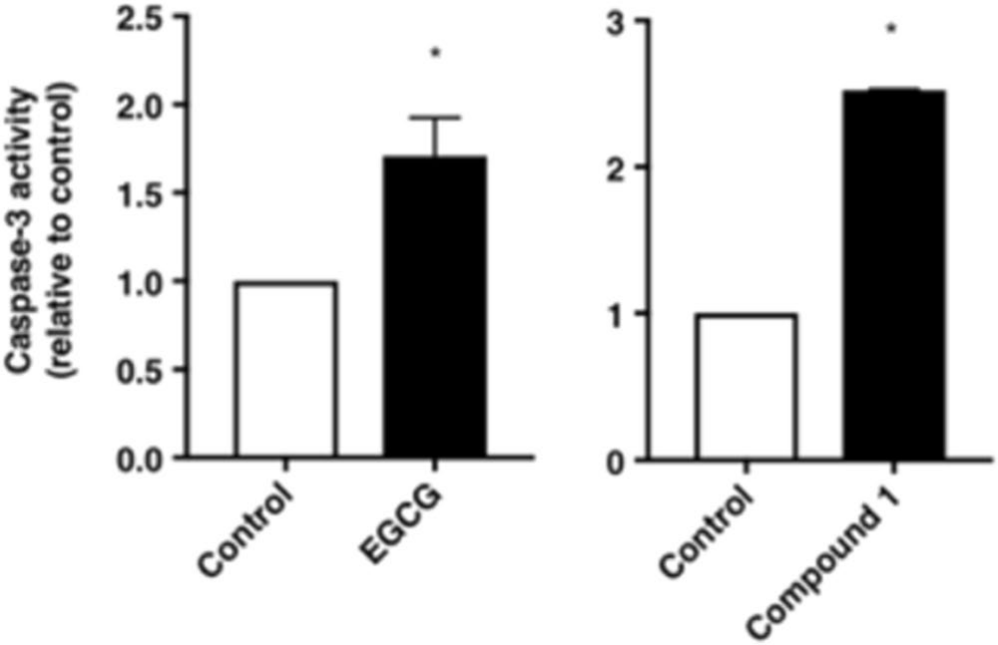
Effects of EGCG and compound 1 on caspase-3 activity in FACS analysis. Caspase 3 activity was evaluated with 5 μmol/L of CPT and 30 μmol/L of EGCG or compound **1**. The graph presents the mean values of FITC fluorescence activity. A shift of the mean FITC fluorescence activity of mock cells to the right side was considered as an increase in apoptosis. The values represent the rate of induction of apoptosis compared with that of the control (vehicle). Data were analysed using Student’s t-test. *P < 0.05.

We next examined the antitumor activities of the newly isolated proanthocyanidin in PC-3 cells (Fig. 2). We first examined effects of compound **1** on PC-3 cell proliferation. EGCG was used as a positive control. Compound **1** showed much higher inhibition of cell growth of PC-3 cells compared with EGCG. Although the observed inhibition on cell growth could be in part attributed to cell cycle arrest (Fig. 3) and induction of apoptosis (Fig. 4), the inhibitory activity of compound **1** against cell proliferation was also observed in colorectal cancer cells (Lovo) and breast cancer cells (MDA-MB-231) in addition to PC-3 cells (data not shown).

### Compound 1 induces cell cycle arrest in PC-3 cells

Considering that the inhibitory activity of compound **1** could be in part attributed to cell cycle arrest or induction of apoptosis, we next examined the effect of compound **1** on cell cycle of PC-3 cells (Fig. 4). FACS analysis showed that treatment of PC-3 cells with EGCG for 48 h induced an increase in the G1 phase population from 61.56% to 76.17% and a decrease in the S phase population from 15.49% to 8.16%. Compound **1** also induced cell cycle arrest at the G1 phase in the PC-3 cells within 48 h. We also investigated whether EGCG or compound **1** suppressed the levels of G1/S phase cell cycle regulators (Cyclin D, Cyclin D3, p21 and c-Myc). Indeed, treatment of PC-3 cells with EGCG or compound **1** for 48 h at a dose of 30 μmol/L significantly decreased these mRNA and protein levels of these regulators compared with the control (data not shown), suggesting that cell cycle arrest at G1/S phase in PC-3 cells.

### Compound 1 induces apoptosis in PC-3 cells

Next, we examined whether suppression of cell proliferation by compound **1** could be attributed to induction of apoptosis. We evaluated the caspase-3 activity as a marker to determine whether cells were undergoing apoptosis. Compound **1** showed higher apoptotic activity in PC-3 prostate cancer cells compared with that of EGCG (Fig. 4).

### Compound 1 suppresses invasive activity of PC-3 cells

**Figure 5:**
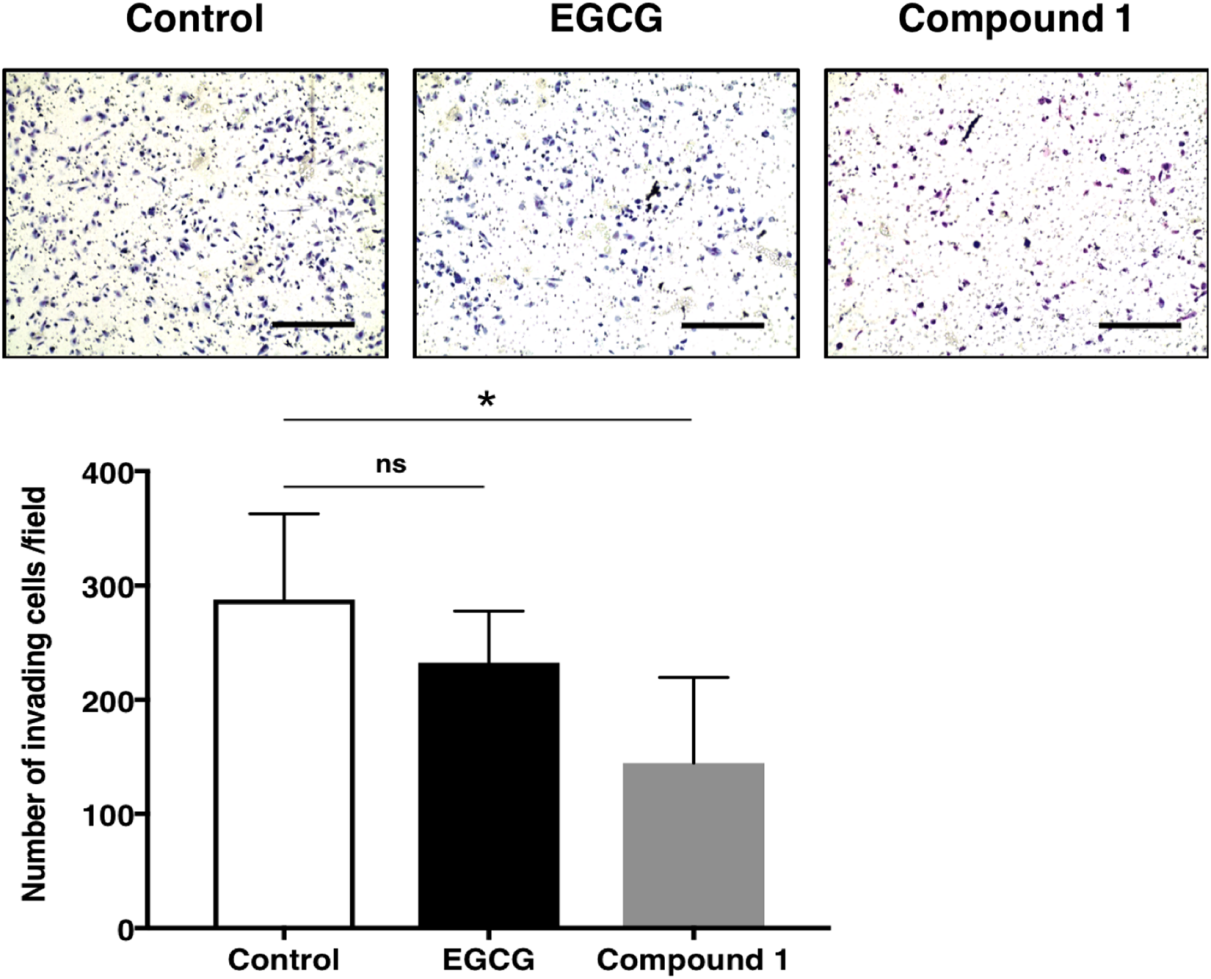
Effects of EGCG and compound 1 on the invasive activity of PC-3 cells. EGCG or compound 1 (30 μmol/L) decreased the invasive activities of PC-3 cells. Representative images of three independent experiments are shown as means ± S.D. of three independent experiments. *P < 0.05, one-way ANOVA followed by Dunnett’s multiple comparison test. Ns, not significant.

**Figure 6:**
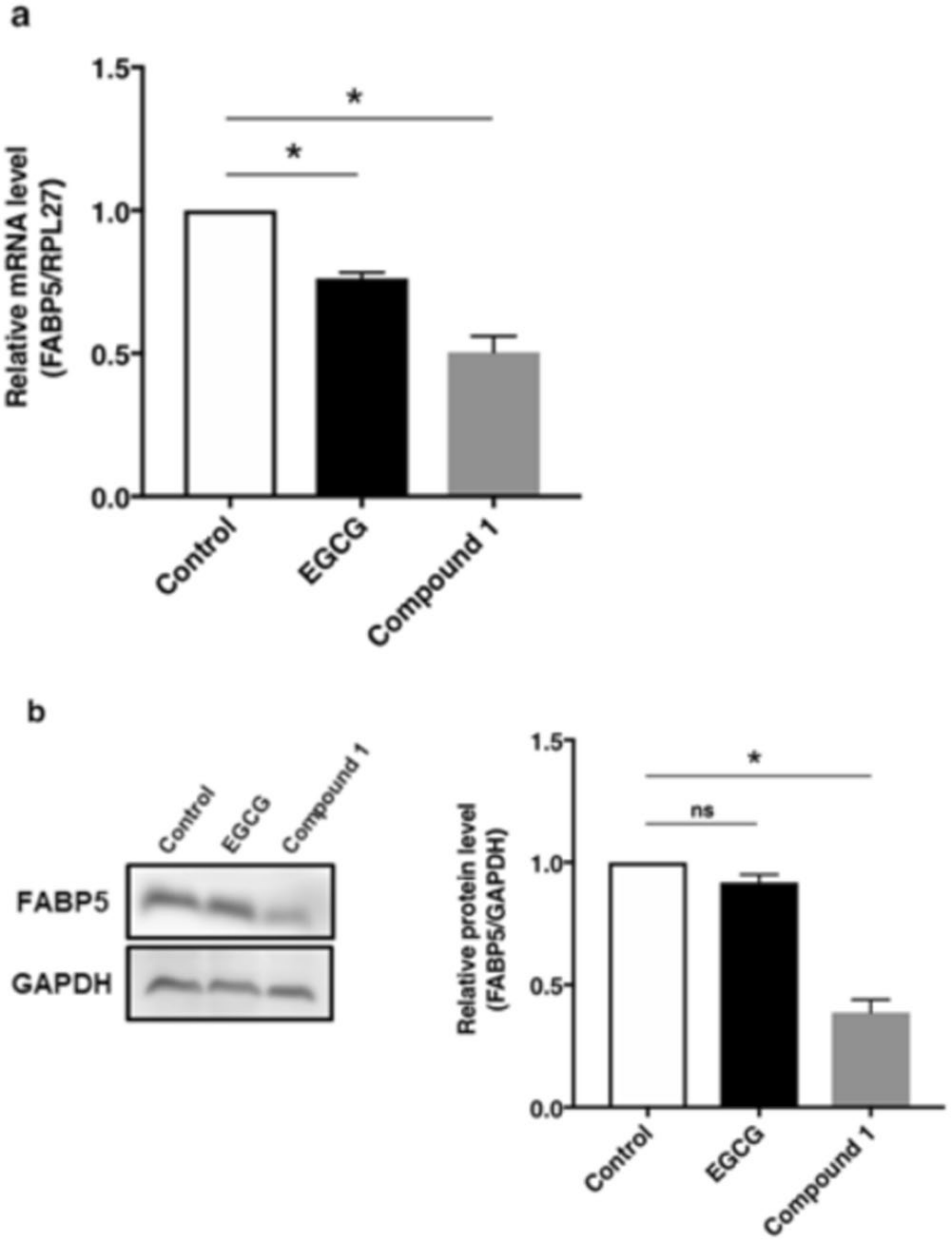
Effects of EGCG and compound 1 on the expression of *FABP5* gene. Compound **1** (30 μmol/L) significantly suppressed *FABP5* gene expression in PC-3 cells. EGCG (30 μmol/L) showed weaker activities than compound **1**. (**a**) Cells treated with EGCG or compound **1** for 48 h were collected and the expression level of FABP5 mRNA was evaluated by qPCR. Relative FABP5 expression levels were quantified and normalized RPL27 levels. The results are shown as the means ± S.D. of three independent experiments. *P < 0.05, one-way ANOVA followed by Dunnett’s multiple comparison test. (**b**) FABP5 protein levels was determined by western blot analysis after treatment of PC-3 cells with EGCG (30 μmol/L) and compound **1** (c.a. 30 μmol/L). Blot data are representative of three independent experiments. Full-length blots are presented in Supplementary Fig. 6. The protein levels of FABP5 were quantified by densitometric measurement using Image J software (NIH). *P < 0.05, one-way ANOVA followed by Dunnett’s multiple comparison test. Ns, not significant.

We next examined the ability of compound **1** to affect PC-3 cell invasion. Compound **1** significantly decreased the number of cells invading through the Matrigel-coated membrane (Fig. 5). This result strongly suggests that compound **1** might suppress the invasiveness of PC-3 prostate cancer cells during metastasis. Suppression of invasive activity by compound **1** was stronger than that of EGCG at 30 μmol/L (Fig. 5). *FABP5* plays an important role in invasion of cancer cells during metastasis, and thus suppression of the invasion activity by these epicatechin oligomers may be partially attributable to down-regulation of *FABP5* gene expression by compound **1** (Fig. 6).

### Compound 1 significantly suppressed the expression of the cancer-promoting gene *FABP5*

We next evaluated the effect of compound **1** on the expression of *FABP5* gene. We previously showed that high expression of the *FABP5* gene plays an important role in cell proliferation and metastasis in various cancer cells^24,25^. More recently, we demonstrated that the *FABP5* gene is epigenetically regulated in human prostate carcinogenesis^24^, supporting its crucial role in tumorigenesis. Moreover, altered fatty acid metabolism is observed in various cancers including prostate cancer. Prostate cancer represents a lipogenic phenotype that utilizes fatty acid oxidation as a dominant bioenergetics pathway to support cell proliferation. *FABP5* might play a pivotal role in fatty acid metabolism as a lipid transporter and/or an important regulatory factor for lipid metabolism in prostate cancer cells. Indeed, we found that *FABP5* knockdown significantly inhibited the gene expressions of fatty acid-metabolizing enzymes and invasive activity of tumour cells^25^. This formed the foundation of our approach to search for compounds with potential anti-cancer activities in grape stem extracts by assessing inhibitory activity on *FABP5* gene expression. As shown in Fig. 6, compound **1** significantly suppressed the gene expression of *FABP5* gene at mRNA and protein levels. EGCG, which possesses only one epicatechin unit, showed weaker gene suppression activities than compound **1**. This was probably because of the differences in the three-dimensional structures of the two compounds, as reported previously^11^. It would be interesting to identify a putative target molecule that interacts with compound **1**, but not with EGCG. Further studies are required to investigate the regulatory mechanisms underlying the suppressive activities of compound **1** on expression of the cancer-promoting genes.

Full images of Western blot analyses of FABP5 protein levels in response to test compounds are shown in Supplementary Fig. 13.

## Discussion

Reports of the isolation and characterization of epicatechin oligomers longer than octamers are quite limited. In the present study we estimated the novel anti-cancer compound as epigallocatechin-(epicatechin)_7_ gallate (compound **1**) from the grape stem extracts using ESI-TOFMS analysis and thiolytic degradation. Compound **1** showed much higher inhibition of cell growth of PC-3 cells compared with EGCG, a well-known cancer preventing agent. Compound **1** also induced cell cycle arrest at the G1 phase and showed high apoptotic activity. Compound **1** also exhibited significantly higher anti-cancer activities than EGCG through suppression of cell growth, the expression of the metastasis-promoting gene *FABP5* and invasion of cancer cells. These results suggested that compound **1** may represent a promising anti-metastatic agent. The oligomeric epicatechin structure is necessary for inhibition of cell proliferation and metastasis, as previously reported^11^. Whether compound **1** interacts with a potent receptor in cancer cells should be examined in future studies.

Our results indicate that novel proanthocyanidin isolated from the grape stems could be potential anti-cancer agents for various cancers. Further mechanistic and *in vivo* studies would be required to confirm these anti-cancer activities.

## Methods

Amberlite XAD-1180N was purchased from ORGANO Corporation, Sephadex LH-20 from GE Healthcare, and Toyopearl HW 40 F from TOSOH Corporation. The stems of *Vitis vifera*, Chardonay, were collected at St. Cousair Winery of Oiri Viniyard in Nagano, Japan in 2012. Catechin tetramers and pentamers, as well as the dimeric to pentameric units of epicatechin were synthesized in our laboratory as standards^1^. EGCG was generously gifted from Dr. Toshiyuki Kan in the University of Shizuoka.

### Statistical analysis

Data obtained in the present study were statistically analyzed by GraphPad Prism 7.03. Data were indicated as the mean ± S.D. One-way analysis of variance (ANOVA) with Turkey’s post-hoc test or two-way ANOVA with Dunnett’s post-hoc test or Student’s t-test was used for multiple comparisons. P < 0.05 was considered to indicate statistical significance.

### Experimental data

The isolation and characterization of a novel oligomeric proanthocyanidin in grape stem are described in Supplementary Information pages 3–31. Biochemical methods were shown in Supplementary Information pages 32–37.

## Supplementary information

Supplementary Information (Isolation and characterization of a novel oligomeric proanthocyanidin with significant anti-cancer activities from grape stems (Vitis vinifera)

## References

[1] Ferreira D, Li X–C (2000). Oligomeric Proanthocyanidins: naturally occurring *O*-heterocycles. Nat. Prod. Rep..

[2] Ferreira D, Slade D (2002). Oligomeric Proanthocyanidins: naturally occurring *O*-heterocycles. Nat. Prod. Rep..

[3] Corder R (2006). Red wine procyanidins and vascular health. Nature.

[4] Terra X (2007). Grape-seed procyanidins act as anti-inflammatory agents in endotoxin-stimulated RAW 264.7 macrophages by inhibiting NF-kB signaling pathway. J. Agric. Food Chem..

[5] Kolodziej H, Haberland C, Woerdenbag HJ, Konings AWT (1995). Moderate cytotoxicity of proanthocyanidins to human tumor cell lines. Phytotherapy Res..

[6] Gall HU, Perchellet FM, Gao XM, Karchesy JJ, Perchellet JP (1994). Comparison of the Inhibitory Effects of Monomeric, Dimeric, and Trimeric Procyanidins on the Biochemical Markers of Skin Tumor Promotion in Mouse Epidermis *in vivo*. Planta Med..

[7] Mitsuhashi S, Saito A, Nakajima N, Shima H, Ubukata M (2008). Pyrogallol Structure in Polyphenols is Involved in Apoptosis-induction on HEK293T and K562 Cells. Molecules.

[8] Kozikowski AP, Tückmantel W, Böttcher G, Romanczyk LJ (2003). Studies in polyphenol chemistry and bioactivity. 4. Synthesis of trimeric, tetrameric, pentameric, and higher oligomeric epicatechin-derived procyanidins having all-4β,8-interflavan connectivity and their inhibition of cancer cell growth through cell cycle arrest. J. Org. Chem..

[9] Fujii W (2013). Syntheses of prodelphinidin B3 and C2, and their antitumor activities through cell cycle arrest and caspase-3 activation. Tetrahedron.

[10] Fujii W (2013). Syntheses of prodelphinidin B1, B2 and B4 and their antitumor activities against human PC-3 prostate cancer cell lines. Tetrahedron Lett..

[11] Takanashi K (2017). Epicatechin oligomers longer than trimers have anti-cancer activities, but not the catechin counterparts. Sci. Rep..

[12] Morgan EA (2008). Expression of cutaneous fatty acid-binding protein (CFABP) in prostate cancer: potential prognostic marker and target for tumourigenicity-suppression. Int. J. Oncol.

[13] Adamson J (2003). High level expression of cutaneous fatty acid-binding protein in prostatic carcinomas and its effect on tumorigenicity. Oncogene.

[14] Oizumi Y, Mohri Y, Hirota M, Makabe H (2010). Synthesis of Procyanidin B3 and Its Anti-inflammatory Activity. The effect of 4-alkoxy group of catechin electrophile in the Yb(OTf)_3_ catalyzed condensation with catechin nucleophile. J. Org. Chem..

[15] Oizumi Y (2012). Synthesis of procyanidin C2 and C1 using Lewis acid mediated equimolar condensation. Heterocycles.

[16] Esatbeyoglu T, Jaschok-Kenter B, Wray V, Winterhalter P (2011). Structure elucidation of procyanidin oligomers by low-temperature ^1^H NMR spectroscopy. J. Agric. Food Chem..

[17] Ucar MB, Ucar G, Pizzi A, Gonultas O (2013). Characterization of *Pinus brutia* bark tannin by MALDI-TOF MS and ^13^C NMR. Industrial Crops and Products.

[18] Mohri Y (2007). An efficient synthesis of procyanidins. Rare earth metal Lewis acid catalyzed equimolar condensation of catechin and epicatechin. Tetrahedron Lett..

[19] Suda M (2013). Syntheses of procyanidin B2 and B3 gallate derivatives using equimolar condensation mediated by Yb(OTf)_3_ and their antitumor activities. Bioorg. Med. Chem. Lett..

[20] Hamauzu Y, Kishida H, Yamazaki N (2018). Gastroprotective property of *Pseudocydonia sinensis* fruit jelly on the ethanol-induced gastric lesions in rats. J. Funct. Foods.

[21] Selga A (2010). Absorption and metabolization of cytoprotective epicatechin thio conjugates in rats. Drug Metab. Dispos..

[22] Wang H, Liu T, Song L, Huang D (2012). Profiles and α-amylase inhibition activity of proanthocyanidins in unripe *Manikara zapota* (Chiku). J. Agric. Food Chem..

[23] Gu L (2003). Screening of foods containing proanthocyanidins and their structural characterization using LC-MS/MS and thiolytic degradation. J. Agric. Food Chem..

[24] Kawaguchi K (2016). The cancer-promoting gene fatty acid-binding protein 5 (FABP5) is epigenetically regulated during human prostate carcinogenesis. Biochem. J..

[25] Kawaguchi K (2016). High expression of fatty acid-binding protein 5 promotes cell growth and metastatic potential of colorectal cancer cells. FEBS Open Bio.

