# Supplementary material for: Isolation and characterization of a novel oligomeric proanthocyanidin with significant anti-cancer activities from grape stems (Vitis vinifera)
Source: Sci Rep. 2019 Aug 19;9:12046. doi: 10.1038/s41598-019-48603-5 (PMC6700121; doi:10.1038/s41598-019-48603-5)
Supplement: Supplementary file 1 — Supplementary Information (Isolation and characterization of a novel oligomeric proanthocyanidin with significant anti-cancer activities from grape stems (Vitis vinifera) [file 41598_2019_48603_MOESM1_ESM.pdf]

Supplementary Information

Isolation and characterization of a novel oligomeric proanthocyanidin with significant anti-cancer activities from grape stems (*Vitis vinifera*)

Sei-ichi, Kawahara † [1,2], Kazuya Toda † [3], Kiriko Matsumoto[4], Chisato Ishihara[4], Shuhei Nonobe[4], Chisato Matsunaga[3], Yoshika K. Gomi[3], Shogo Senga[1], Koichiro Kawaguchi[1], Ayaka Yamamoto[4], Jutalak Suwannachot[5], Reiko, Noda[5], Shuhei Kobayashi[3], Yasunori Hamauzu[1,5], Hidefumi Makabe[1,5,6]*, and Hiroshi Fujii[1,4,6]*

[1]*Interdisciplinary Graduate School of Science and Technology, Shinshu University, 8304 Minami-minowa Kami-ina, Nagano, 399-4598, Japan*

[2]*St. Cousair Co., Ltd., 1260 Imogawa, Kami-minochi, Iizuna, Nagano, 389-1201, Japan*

[3]*Department of Bioscience and Biotechnology, Faculty of Agriculture, Shinshu University, 8304 Minami-minowa Kami-ina, Nagano, 399-4598, Japan*

[4]*Graduate School of Science and Technology, Department of Biomedical Engineering, Shinshu University, 8304 Minami-minowa Kami-ina, Nagano, 399-4598, Japan*

[5]*Graduate School of Science and Technology, Department of Agriculture, Division of Food Science and Biotechnology, Shinshu University, 8304 Minami-minowa Kami-ina, Nagano, 399-4598, Japan*

[6]*Department of Interdisciplinary Genome Sciences and Cell Metabolism, Institute for Biomedical Sciences, Interdisciplinary Cluster for Cutting Edge Research*, *Shinshu University, Minami-minowa, Kami-ina, Nagano*, *399-4598, Japan*

*Corresponding authors: Hidefumi Makabe, Tel. +81 265 77 1630; fax +81 265 77 1700; e-mail: makabeh@shinshu-u.ac.jp;

Hiroshi Fujii, Tel. +81 265 77 1626; fax +81 265 77 1626; e-mail: hfujii@shinshu-u.ac.jp

† contributed equally to this work.

Table of contents

Materials and methods (page 5)

Supplementary Figure 1: HPLC chromatogram of crude hot water extracts of GSE (page 6).

Supplementary Figure 2: Purification scheme of grape stem extracts (page 7).

Supplementary Figure 3: HPLC chromatogram of EtOAc-eluted fraction using Amberlite XAD-1180N resin (page 8).

Supplementary Figure 4: HPLC chromatogram of MeOH-eluted fraction using Amberlite XAD-1180N resin (page 9).

Condition of HPLC method for supplementary Figure 3 and 4 (page 10).

Supplementary Figure 5: Effects of test samples (organic solvent-eluted fractions in Sephadex LH-20 (LH20-30% methanol (MeOH), ethanol (EtOH) and 60% acetone), Toyopearl HW-40F (HW40F-60% acetone, called as fraction 3; Fr. 3) or compound **1**) on the expression of the cancer-promoting gene *FABP5* (page 11).

Supplementary Figure 6: HPLC chromatogram of 60% acetone-eluted fraction (fraction 3) using Toyopearl HW40F resin (page 12).

Condition of HPLC method for supplementary Figure 6 (page 13).

Supplementary Figure 7: ESI-TOFMS spectrum 60% acetone-eluted fraction (fraction 3) using Toyopearl HW40F resin (page 14).

Supplementary Figure 8: HPLC chromatogram of compound **1** purified with reverse phase HPLC (page 15).

Condition of HPLC method for supplementary Figure 8 (page 16).

Supplementary Figure 9a: ^{1}H NMR spectra of compound **1** (page 16-17).

Supplementary Figure 9b: ^{13}C NMR spectra of compound **1** (page 18).

Experimental procedure for thiolytic degradation of compound **1** and LC-MS analysis (page 19)

Supplementary Figure 10a: MS spectra of peak 1 (epigallocatechin-cysteamine thioether, MW = 381) and peak 2 (catechin-cysteamine thioether, MW = 365) in Figure 1 (page 20).

Supplementary Figure 10b: MS spectra of peak 3 (epigallocatechin-gallate-cysteamine thioether, MW = 533) and peak 4 (epicatechin-cysteamine thioether, MW = 365) in Figure 1 (page 21).

Supplementary Figure 10c: MS spectra of peak 5 (catechin, MW = 290) and peak 6 (epicatechin-gallate-cysteamine thioether, MW = 517) in Figure 1 (page 22).

Supplementary Figure 10d: MS spectra of peak 7 (catechin-gallate-cysteamine thioether, MW = 517) and peak 8 (epicatechin, MW = 290) in Figure 1 (page 23).

Supplementary Figure 10e: MS spectra of peak 9 (epicatechin-gallate, MW = 442) and peak 10 (catechin-gallate, MW = 442) in Figure 1 (page 24).

Supplementary Figure 10f: HPLC chromatogram of thiolytic degraded products of GSE (compound 1), epicatecin (EC) dimer, catechin (C) dimer, epicatechin-gallate (ECg) dimer and epigallocatechin (EGC) dimer. '-cys' means cysteamine adduct corresponds to the extension (upper) unit of a dimer (page 25).

HPLC condition and ESI-LC-TOFMS/MS for supplementary Figure 11 and 12 (page 26).

Supplementary Figure 11: ESI-TOF MS spectrum of compound **1** (page 27).

Supplementary Figure 12: ESI-MS/MS spectra of compound **1** with 30-60V (page 28-31).

Supplementary Figure 13: Full images of Western blot analyses of the expression of the cancer-promoting gene *FABP5* by treatment of test compounds (page 32).

Biochemical methods (page 33-37)

Supplementary references (page 37)

Materials and methods

Materials

Amberlite XAD-1180N was purchased from ORGANO Corporation, Sephadex LH-20 from GE Healthcare, and Toyopearl HW 40F from TOSOH Corporation. The stems of *Vitis vifera*, Chardonay, were collected at St. Cousair Winary of Oiri Viniyard in Nagano, Japan in 2012. Catechin tetraners and pentamers, as well as the dimeric to pentameric units of epicatechin were synthesized in our laboratory as standards[1]. EGCG was generously gifted from Dr. Toshiyuki Kan in the University of Shizuoka.

Isolation and characterization of proanthicyanidins from *Vitis vifera*, "Chardonay"

Extraction of proanthocyanidins from *Vitis vifera*, "Chardonay".

One kilogram of fresh stems of *Vitis vifera*, Chardonay, were finely cut and extracted with water (4 L) under reflux for 1 h. The mixture was filtered and the solvent was concentrated *in vacuo* to yield a slurry. The slurry was loaded into Amberlite XAD-1180N resin (1.5 L) and eluted with ethyl acetate (4.5 L) and methanol (4.5 L). The methanol fraction was evaporated to give 3.1 g of crude extract. 1.0 g of this material was chromatographed over Sephadex LH-20 (100 mL) eluting with 30% MeOH, 40% EtOH, 30% acetone, 40% acetone and 60% acetone sequentially to give five fractions. Fraction five (345 mg) was further purified over Toyopearl HW40F (63 mL) eluting with 30% MeOH, 40% EtOH and 60% acetone sequentially to afford three fractions. Fraction three was evaporated to yield a light brown powder (216 mg) for further characterization (Supplementary Figure 1).

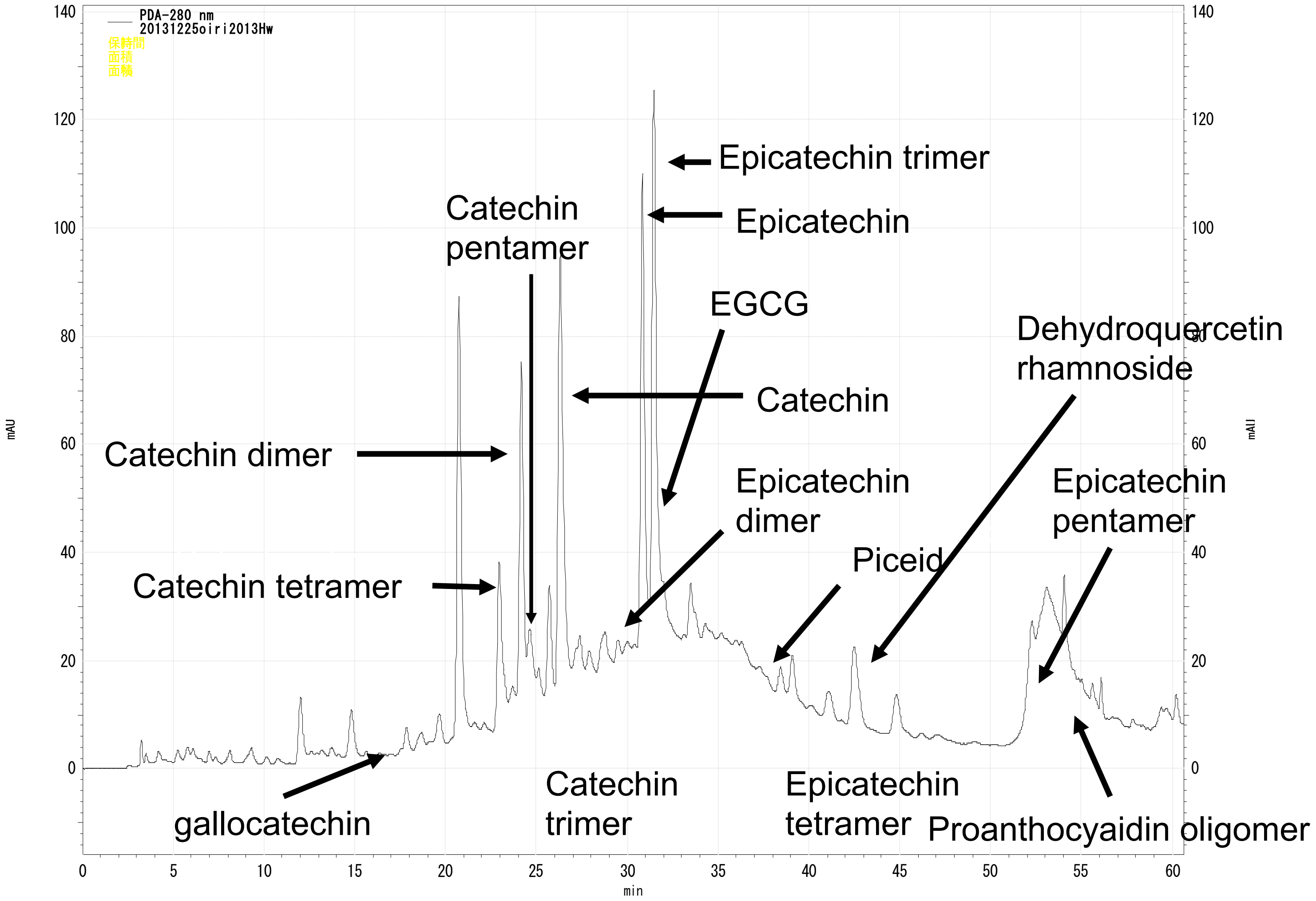

Supplementary Figure 1 | HPLC chromatogram of crude hot water extracts of grape stem extracts (GSE).

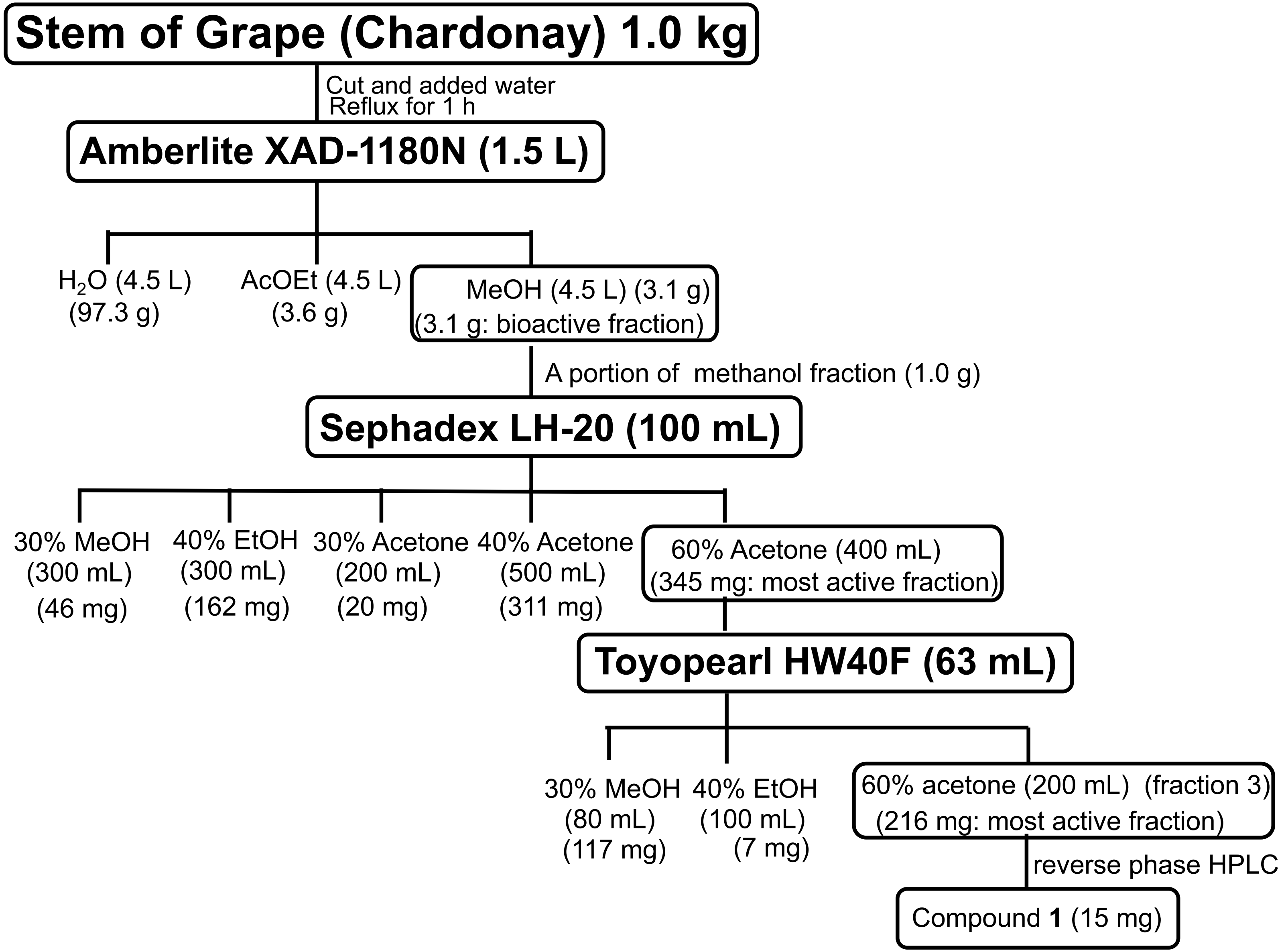

Supplementary Figure 2 | Purification scheme of grape stem extracts (GSE).

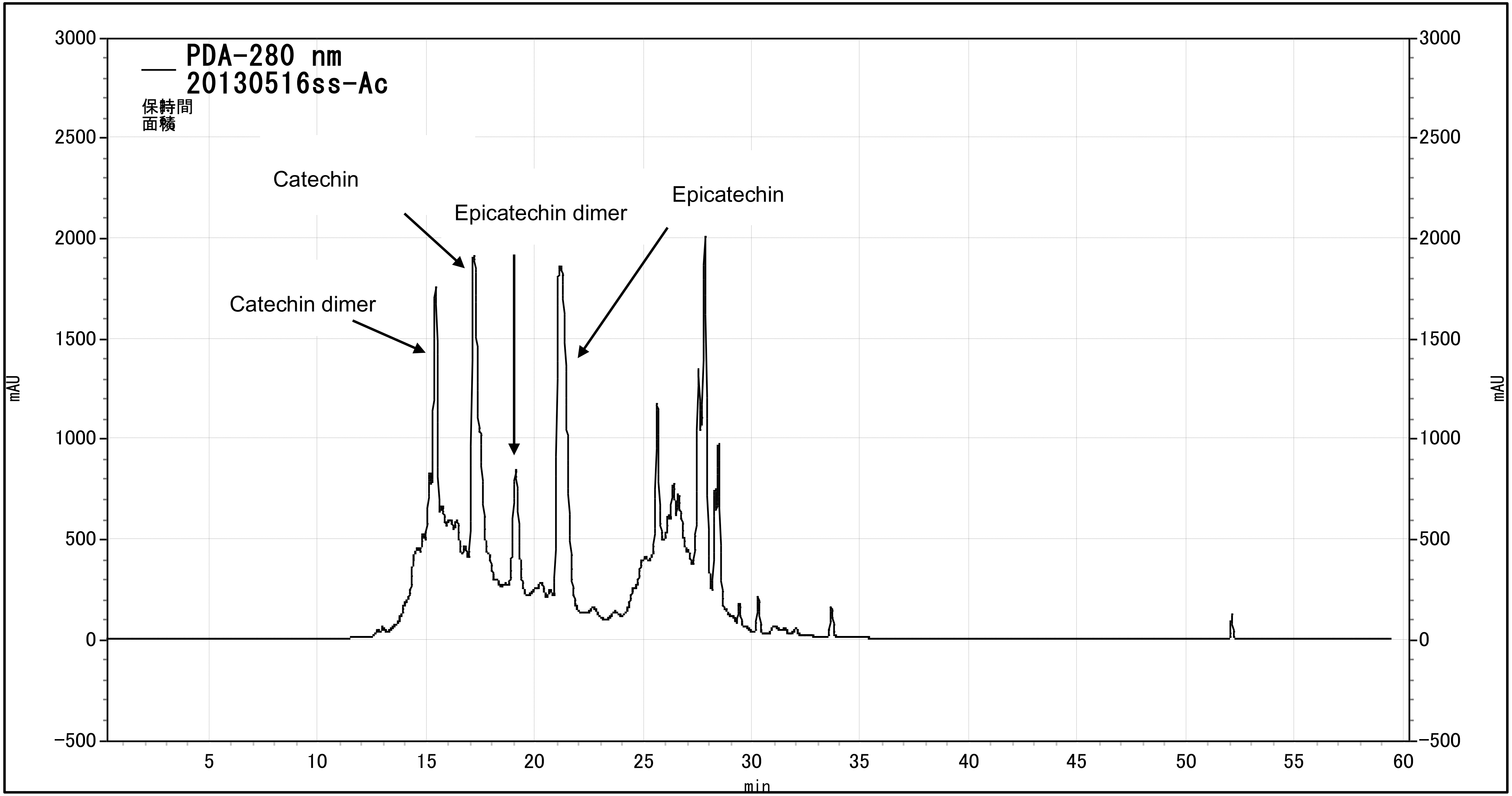

Supplementary Figure 3 | HPLC chromatogram of EtOAc-eluted fraction using Amberlite XAD-1180N resin.

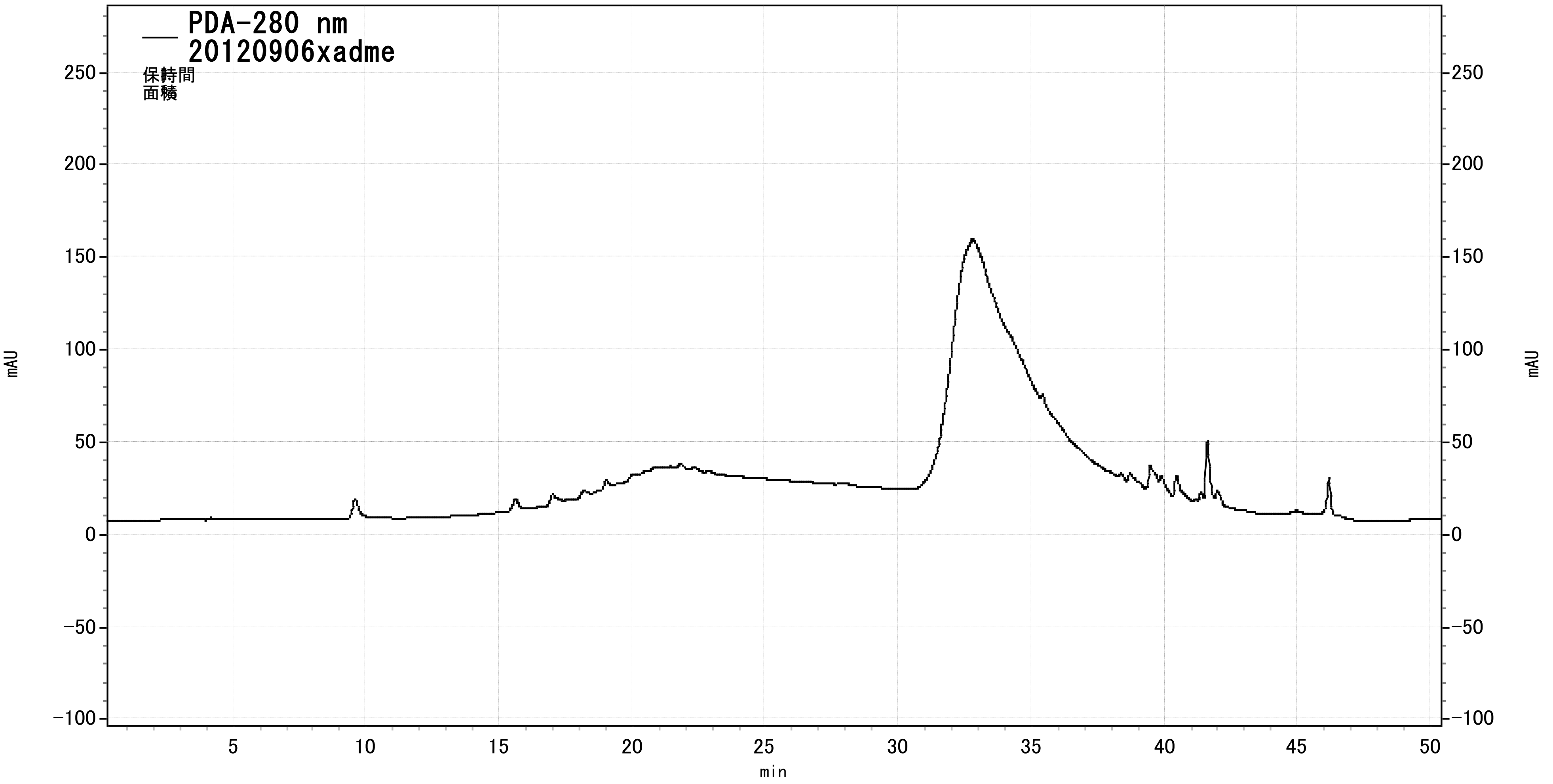

Supplementary Figure 4 | HPLC chromatogram of MeOH-eluted fraction using Amberlite XAD-1180N resin.

Condition of HPLC method for Supplementary Figures 3 and 4.

Retention time (min)	MeOH (V/V %)	H_2O (V/V %)
0 to 5	5	95
5 to 14	25	75
14 to 18	25	75
18 to 25	95	5
25 to 38	100	0
38	100	0

The column temperature was 40°C. The flow rate was 0.8 mL/min. Inertsil ODS-3 column (3 µm, 250 mm x 4.6 mm i,d.)

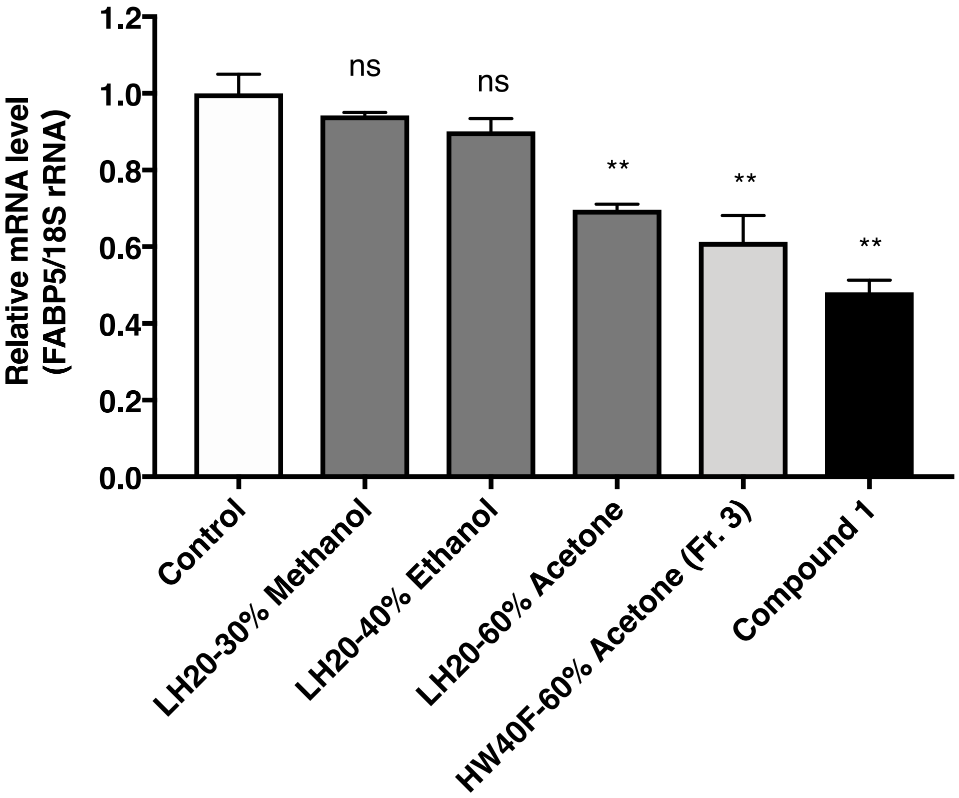

**P<0.01

Supplementary Figure 5 | Effects of test samples (organic solvent-eluted fractions in Sephadex LH-20 (LH20-30% methanol (MeOH), ethanol (EtOH) and 60% acetone), Toyopearl HW-40F (HW40F-60% acetone, called as fraction 3; Fr. 3) or compound **1**) on the expression of the cancer-promoting gene *FABP5*. Concentration of each fraction for assay was 10 μg/μl. Cells were plated in 6-well plates and allowed to reach 50% confluent growth. The cells were treated with the indicated test compounds for 48 h. Experimental procedure for bioassay by qPCR was carried out as described in Supplemental Information. Grape stem extracts were purified by the scheme in Supplementary Figure 2. The MeOH-eluted fraction in Amberlite XAD-1180N chromatography which was the most active fraction was applied to Superdex LH-20 chromatography. The most active fraction, 60% acetone-eluted fraction in Superdex LH-20 chromatography, was evaluated for bioactivity assessed by qPCR and was further purified by Toyopearl HW40 chromatography. The most active fraction, 60% acetone-eluted fraction (fraction 3) in Toyopearl HW40 chromatography was evaluated for bioactivity assessed by qPCR. Fraction 3 was further purified by reverse phase HPLC to get compound **1** and compound **2**.

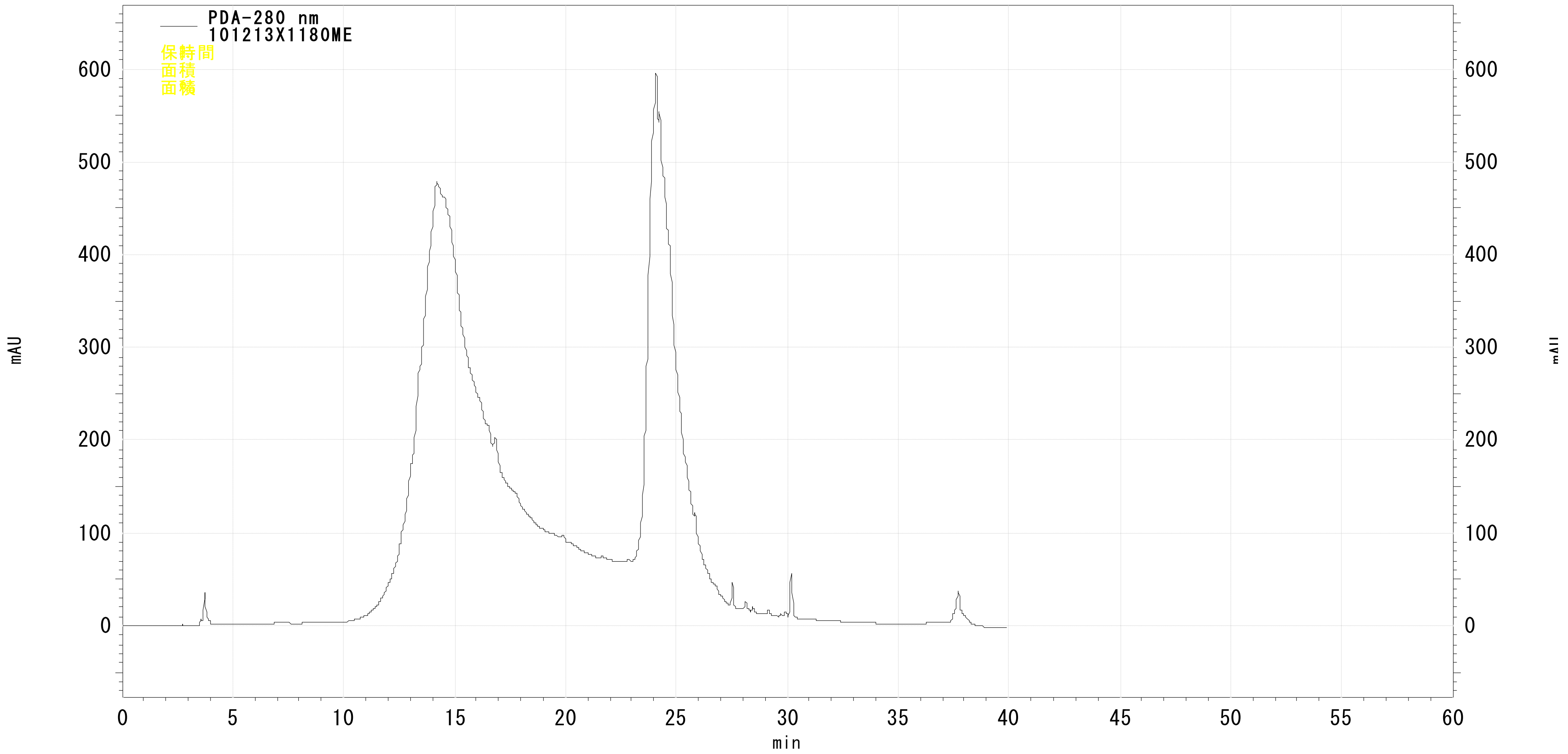

Supplementary Figure 6 | HPLC chromatogram of 60% acetone-eluted fraction (fraction 3) using Toyopearl HW40F resin.

Condition of HPLC method for supplementary Figure 6.

Retention time (min)	MeOH (V/V %)	0.1% AcOH (V/V %)
0 to 5	5	95
5 to 14	25	75
14 to 18	25	75
18 to 25	95	5
25 to 38	100	0
38	100	0

The column temperature was 40°C. The flow rate was 0.8 mL/min. Inertsil ODS-3 column (3 μm, 250 mm x 4.6 mm i,d.)

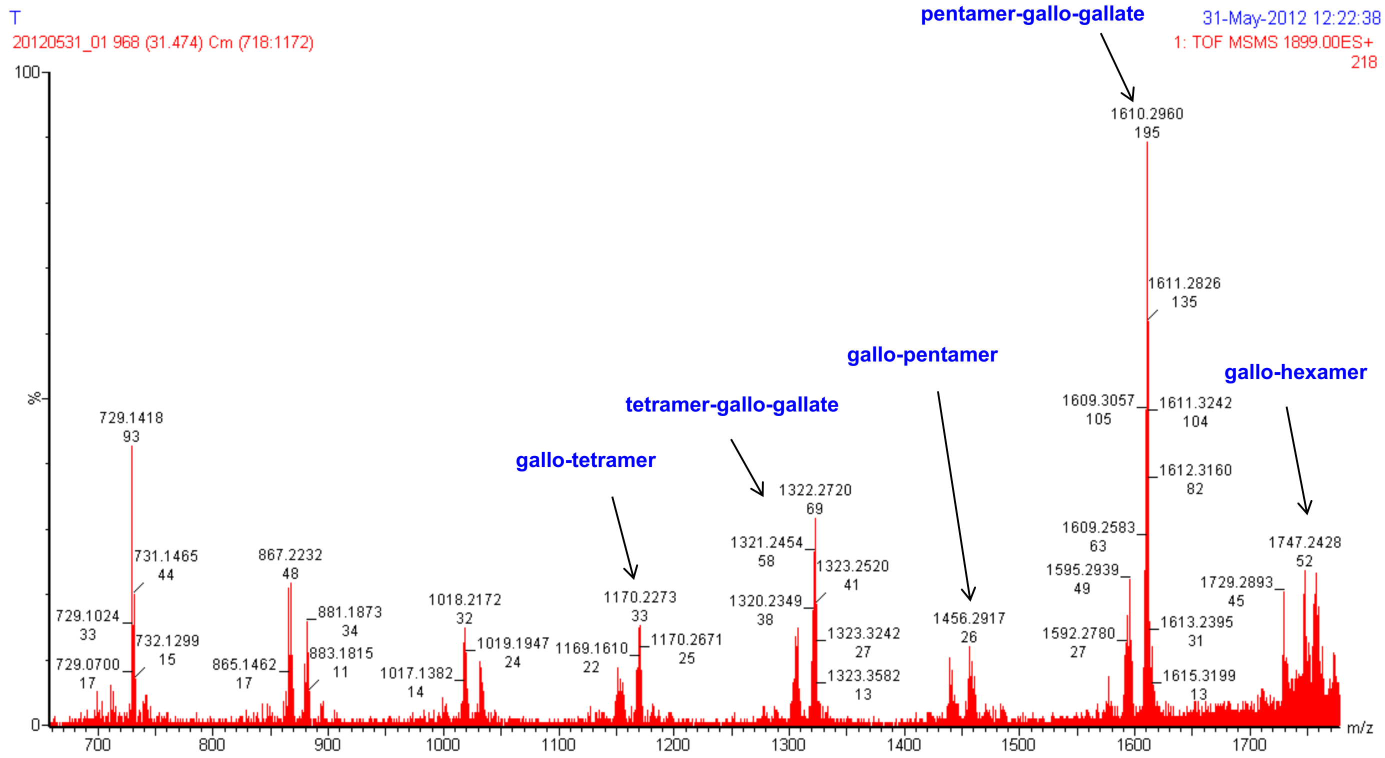

Supplementary Figure 7 | ESI-TOFMS spectrum 60% acetone-eluted fraction (fraction 3) using Toyopearl HW40F resin.

HPLC condition for supplementary Figure 7.

The crude sample of fraction 3 was diluted in MeCN (10 mg/ mL) and filtered through a 0.45 μm PTFE membrane filter prior to injection. 20 μL of sample was injected into an InertSustain C18 column (3 μm, 250 mm x 4.6 mm i,d.) and eluted with mobile phase A (0.1% formic acid) and B (0.1% formic acid in MeCN). The flow rate was 0.5 mL/min. The column temperature was 40 ^{o}C. A gradient elution at 10 min intervals was performed as follows: 0-10 min: 90% A and 10% B, 11-49 min 90% A and 10% B, 50-59 min: 50% A and 50% B, 60 min: 100% B.

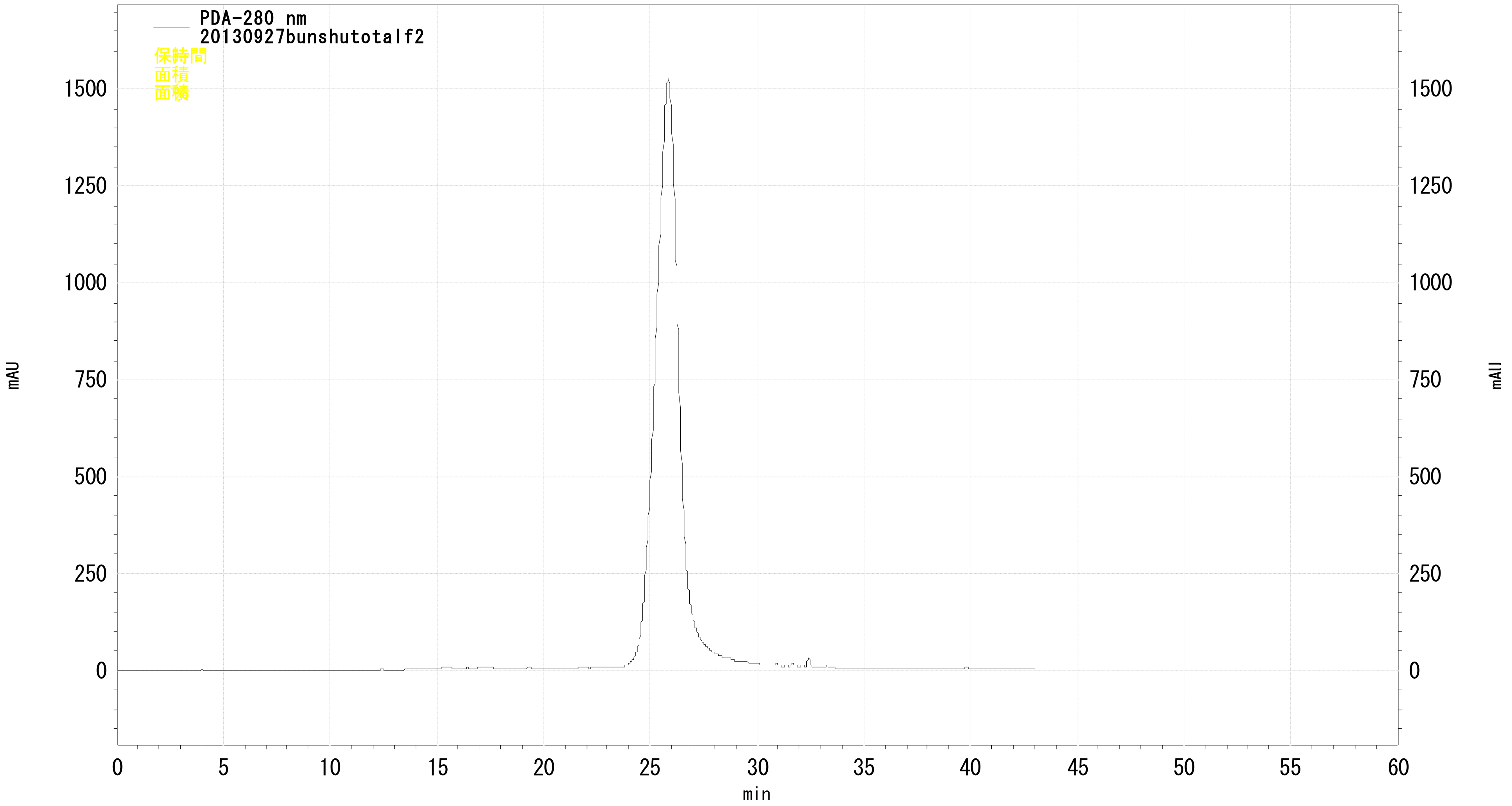

Supplementary Figure 8 | HPLC chromatogram of compound **1** purified with reverse phase HPLC.

Condition of HPLC method for supplementary Figure 8.

Retention time (min)	MeOH (V/V %)	0.1% AcOH (V/V %)
0 to 5	5	95
5 to 14	25	75
14 to 18	25	75
18 to 25	95	5
25 to 38	100	0
38	100	0

The column temperature was 40°C. The flow rate was 0.8 mL/min. Inertsil ODS-3 column (3 μm, 250 mm x 4.6 mm i,d.)

NMR

^{1}H and ^{13}C NMR spectra were recorded on Bruker Avance 500 MHz spectrometer. Chemical shifts were reported in ppm on the δ scale relative to tetramethylsilane (TMS) (δ = 0.00 for ^{1}H NMR), CD_3OD (δ = 49.0 for ^{13}C NMR) as internal references.

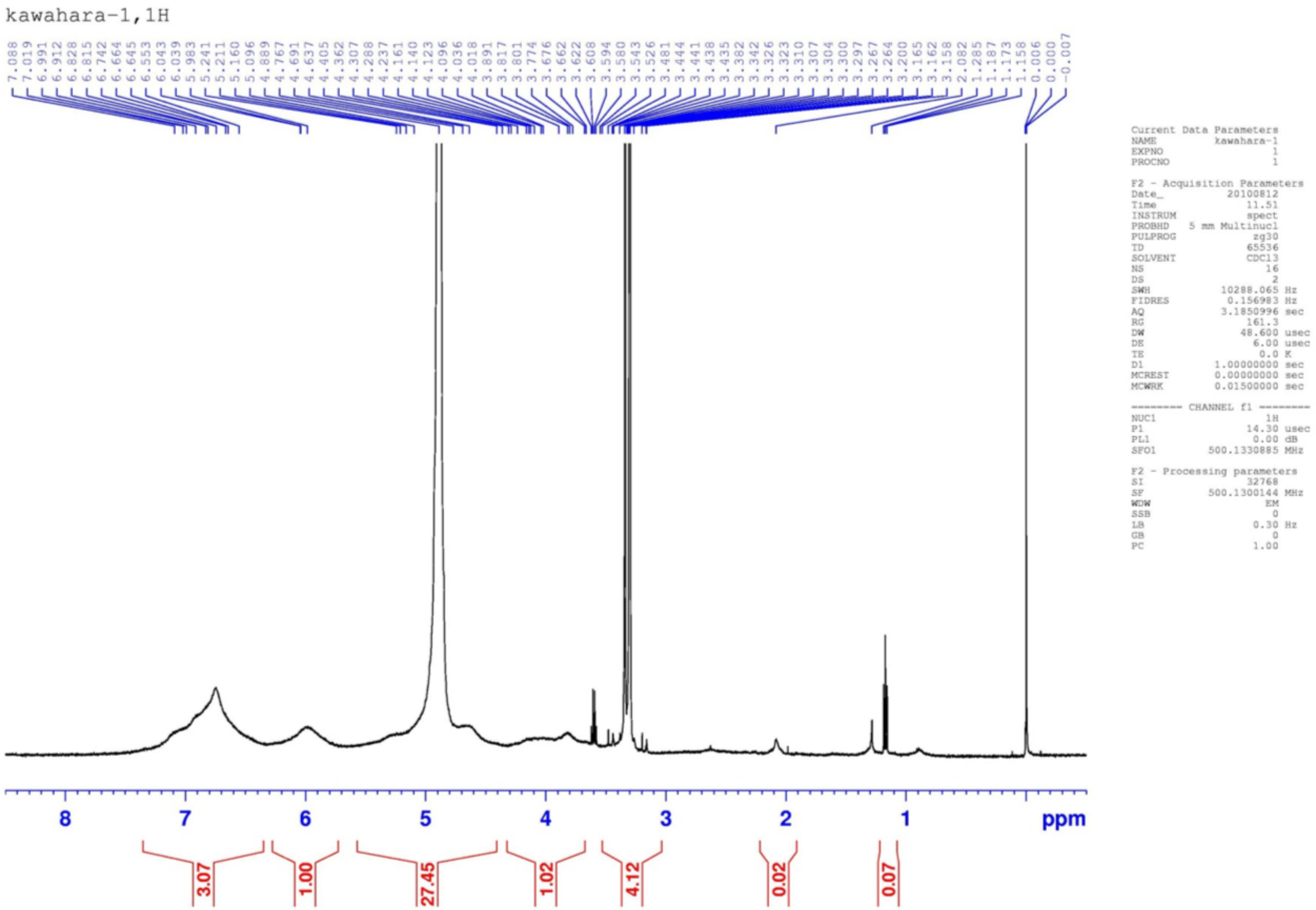

Supplementary Figure 9a | ^{1}H NMR spectra of compound **1**.

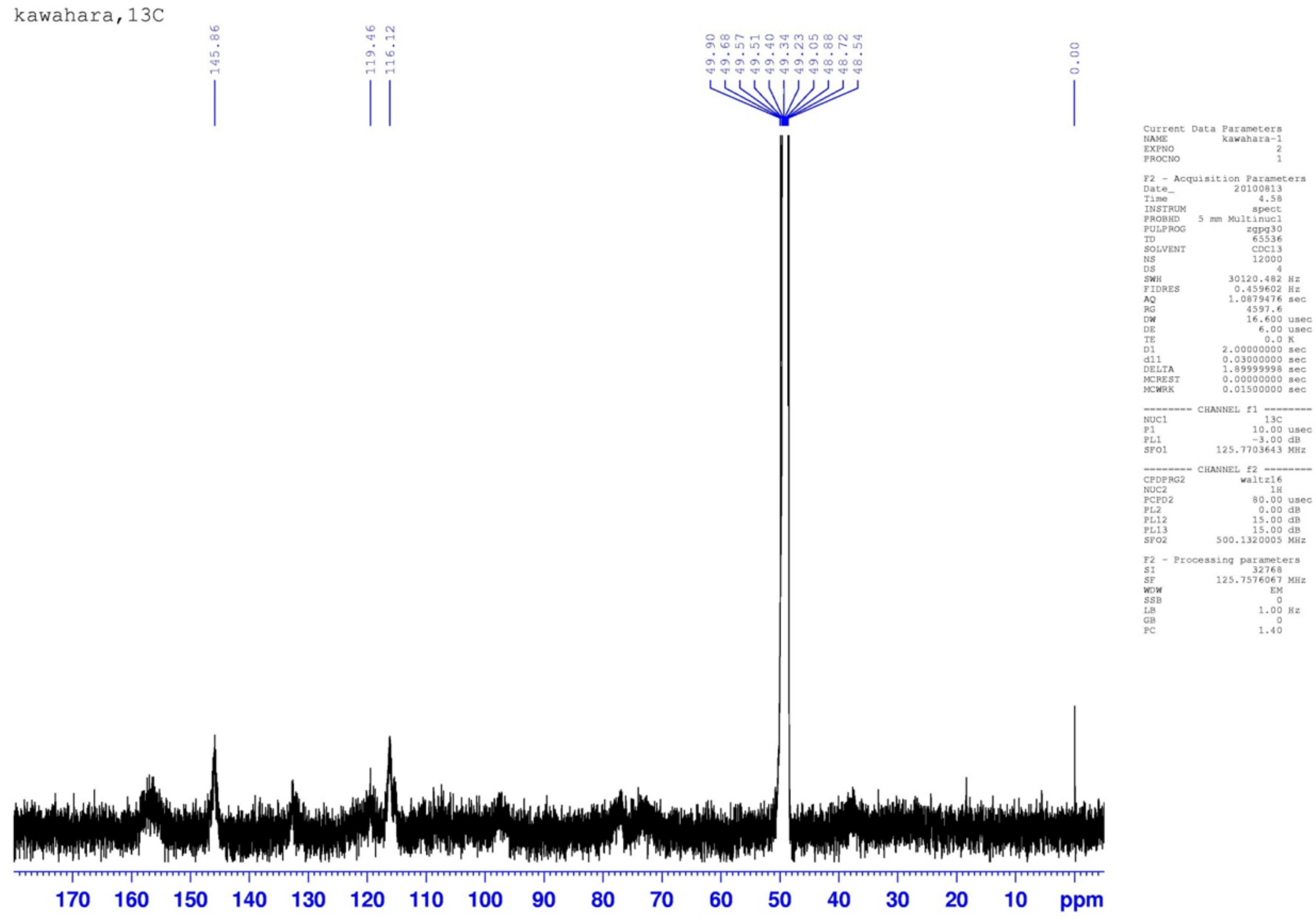

Supplementary Figure 9b | ^{13}C NMR spectra of compound **1**.

Thiolytic degradation of compound 1 and LC-MS analysis

Constitutional flavan-3-ol units of GSE were analyzed using HPLC-DAD-ESI-MS system after thiolytic degradation, that was performed according to Hamauzu et al. (2018)[2] with some modifications. In a screw-cap test tube, an aliquot (200 µL) of GSE methanolic solution was mixed with a 200 µL of 10% (w/v) 2-mercaptoethylamine hydrochloride (cysteamine hydrochloride) methanolic solution containing 0.3 mol/L HCl was added and mixed. The mixture was incubated for 30 min at 75 °C. To minimize the isomerization, incubation 90 min at 50˚C was also used. The reaction mixture was diluted with twice its volume of water, filtered using 0.45-µm membrane filter and analyzed using ACQUITY UPLC separation module coupled with a Waters Micromass Q-micro MS(/MS) system that was controlled with a MassLynx v4.1 data analysis software (Waters, Milford, MA, USA). The column used was a Phenomenex Luna C_{18} (150 x 4.6 mm, 5 µm) with a security guard cartridge (4.0 x 3.0 mm). The solvents used were: (A) 0.5% (v/v) formic acid in 5% (v/v) acetonitrile, and (B) 0.5% (v/v) formic acid/20% (v/v) 2-propanol in acetonitrile. The gradient program was initiated with 0% solvent B (0–1 min), and the gradient was slowly shifted to obtain 5% solvent B at 10 min, 5% B at 15 min, 40% B at 40 min, and 90% B at 50 min. The flow rate was 0.5 mL/min. The mass spectrometer was operated in negative ion mode to analyze thiolytic degraded mixture in the following conditions: capillary voltage of −3.0 kV and nebulizer gas (N_2) temperature of 350˚C (gas flow of 600 L/h). The cone voltage was set 25 V. The mass spectra were scanned over the m/z range 100 – 1000. Analysis in the positive ion mode was also conducted for confirmation. Thiolysis of proanthocyanidin dimers, i.e. individual dimers of (–)-epicatechin, (+)-catechin, (–)-epicatechin-gallate and (–)-epigallocatechin, was also conducted to confirm retention time of cysteamine adducts of each catechin monomer.

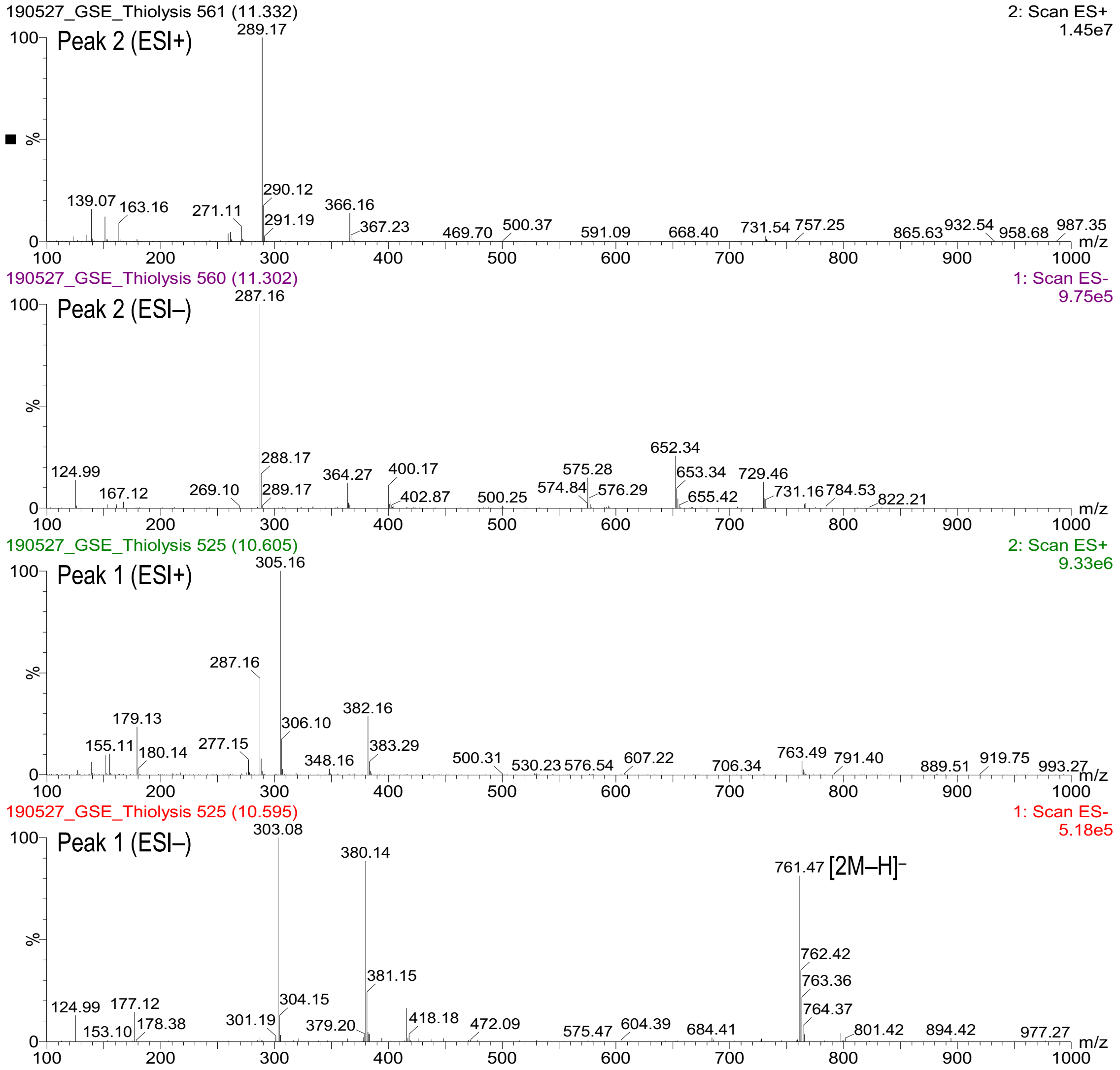

Supplementary Figure 10a | MS spectra of peak 1 (epigallocatechin-cysteamine thioether, MW = 381) and peak 2 (catechin-cysteamine thioether, MW = 365) in Figure 1.

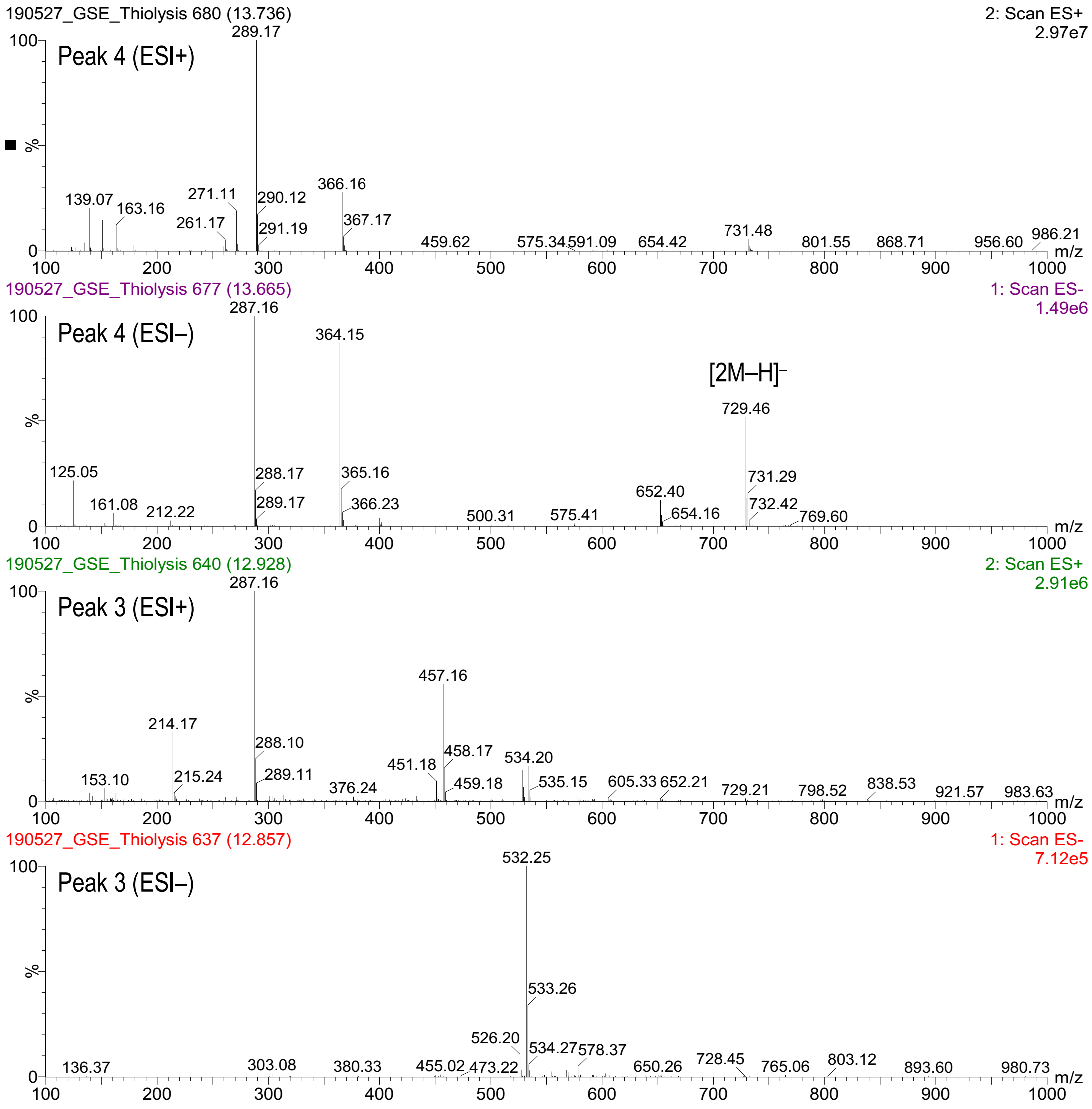

Supplementary Figure 10b | MS spectra of peak 3 (epigallocatechin-gallate-cysteamine thioether, MW = 533) and peak 4 (epicatechin-cysteamine thioether, MW = 365) in Figure 1.

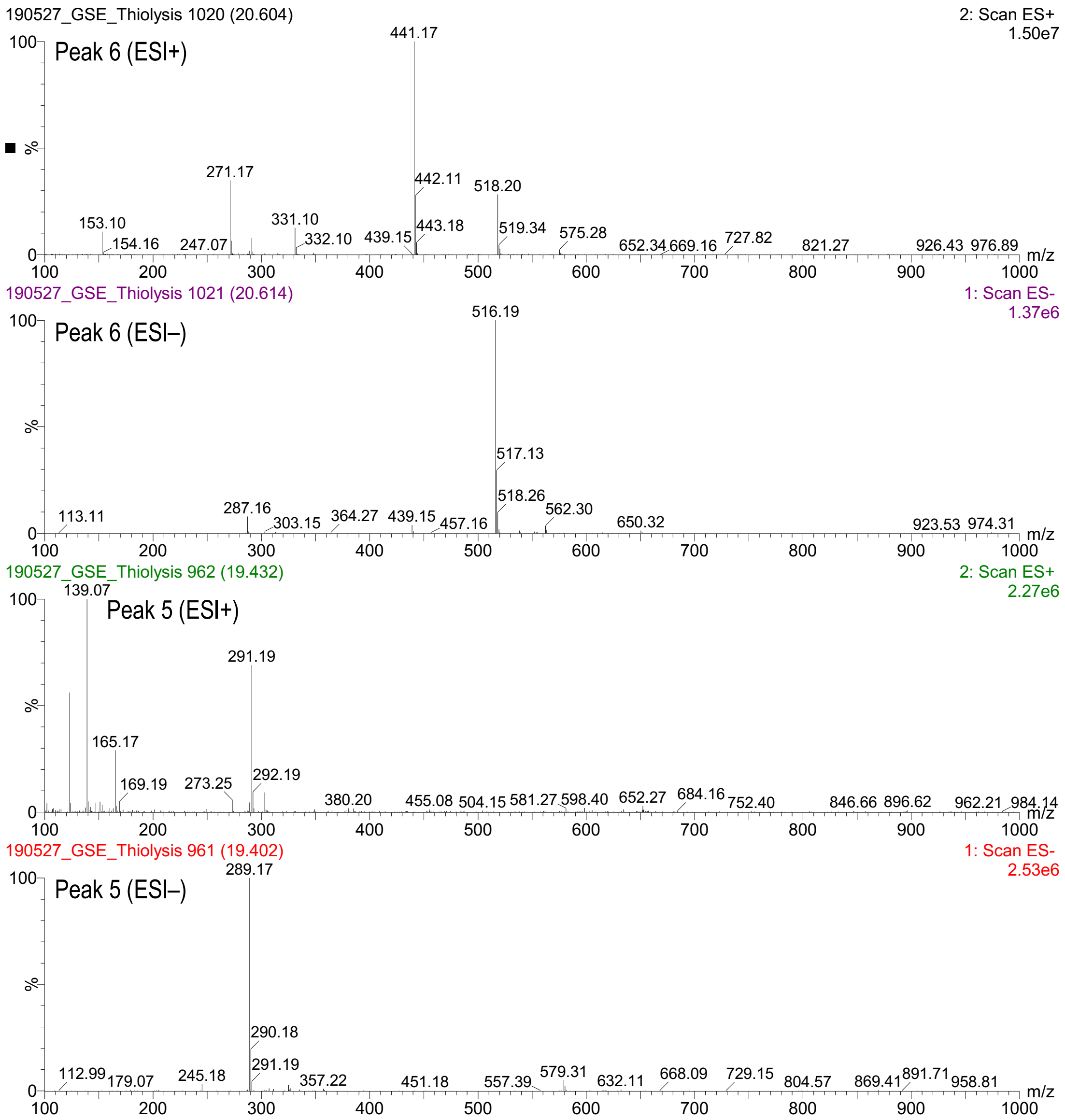

Supplementary Figure 10c | MS spectra of peak 5 (catechin, MW = 290) and peak 6 (epicatechin-gallate-cysteamine thioether, MW = 517) in Figure 1.

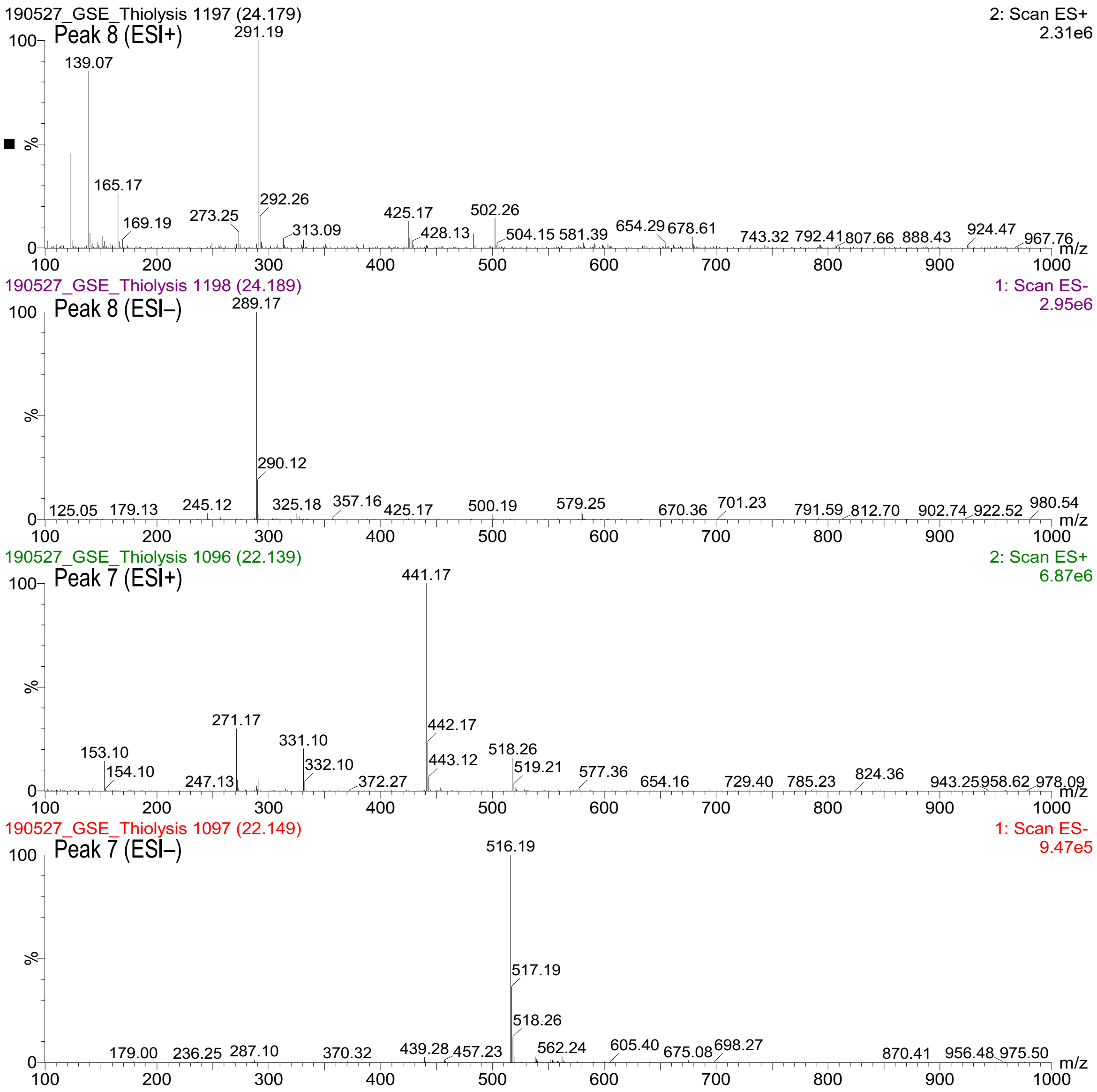

Supplementary Figure 10d | MS spectra of peak 7 (catechin-gallate-cysteamine thioether, MW = 517) and peak 8 (epicatechin, MW = 290) in Figure 1.

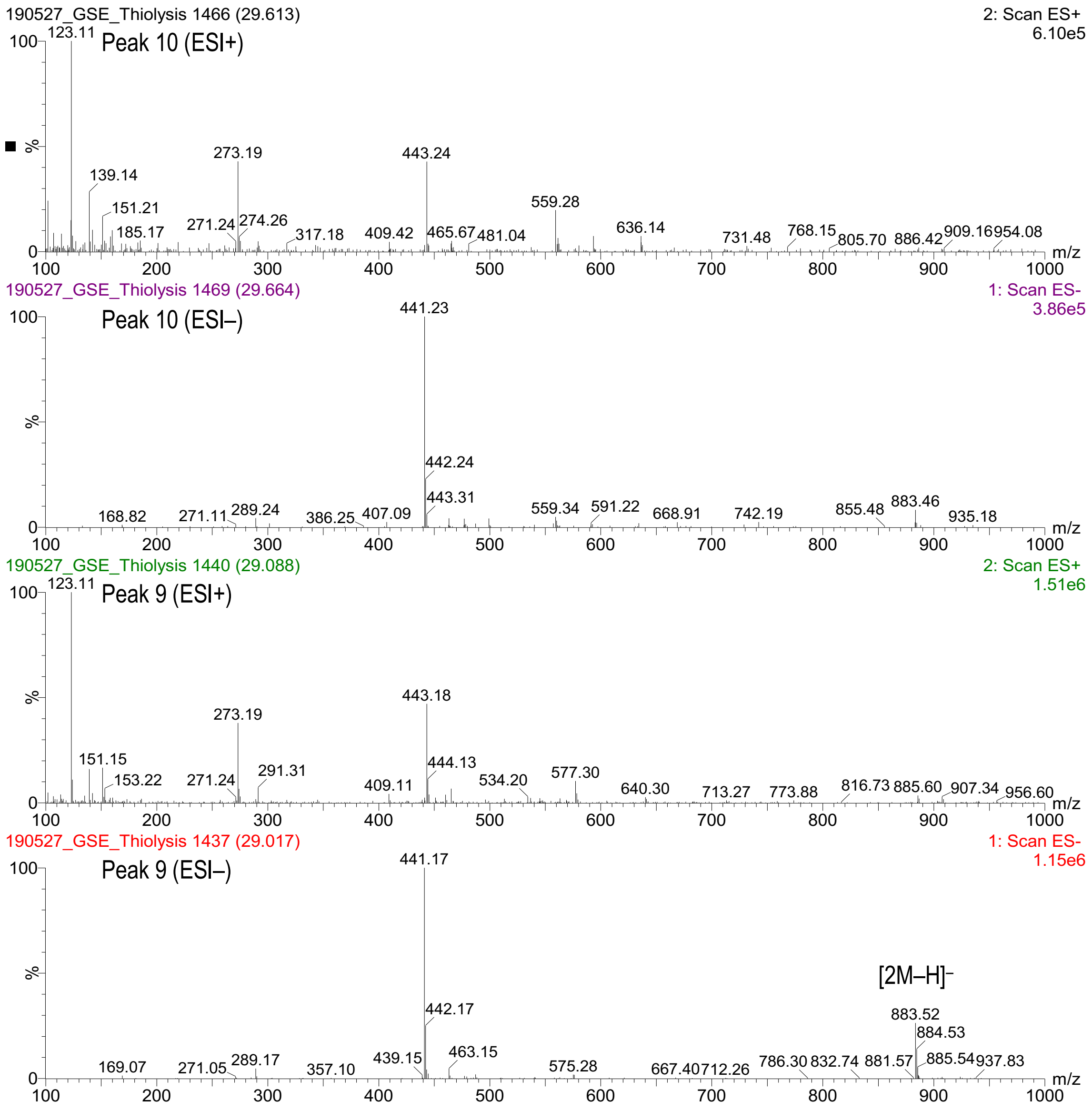

Supplementary Figure 10e | MS spectra of peak 9 (epicatechin-gallate, MW = 442) and peak 10 (catechin-gallate, MW = 442) in Figure 1.

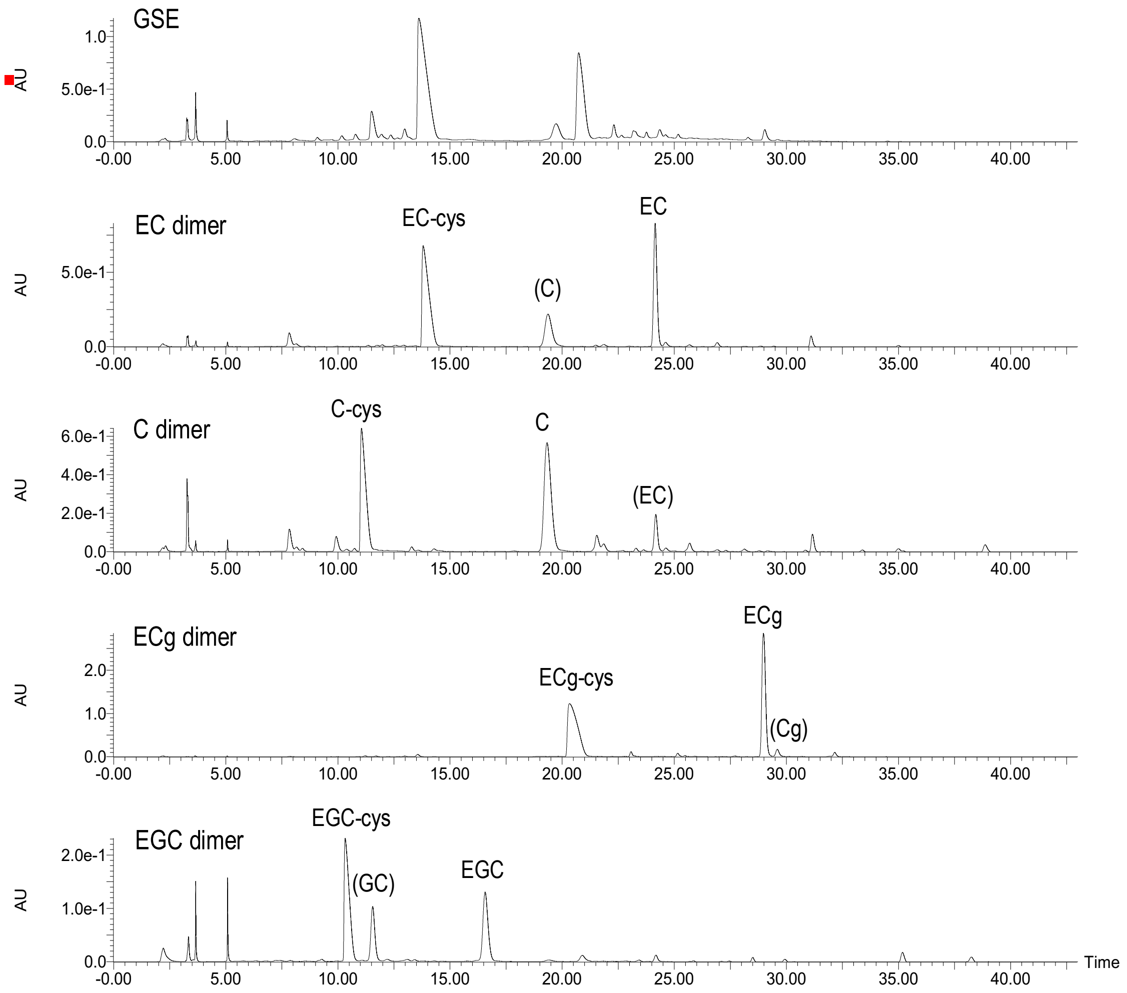

Supplementary Figure 10f | HPLC chromatogram of thiolytic degraded products of GSE (compound 1), epicatecin (EC) dimer, catechin (C) dimer, epicatechin-gallate (ECg) dimer and epigallocatechin (EGC) dimer. '-cys' means cysteamine adduct corresponds to the extension (upper) unit of a dimer. Peak label in a parenthesis represents a product by isomerization of terminal (lower) unit occurred during thiolysis.

HPLC condition for supplementary Figure 11 and 12.

The crude sample of fraction 3 was diluted in MeCN (10 mg/ mL) and filtered through a 0.45 µm PTFE membrane filter prior to injection. 20 µL of sample was injected into an InertSustain C18 column (3 µm, 250 mm x 4.6 mm i,d.) and eluted with mobile phase A (0.1% formic acid) and B (0.1% formic acid in MeCN). The flow rate was 0.5 mL/min. The column temperature was 40°C. A gradient elution at 10 min intervals was performed as follows: 0-10 min: 90% A and 10% B, 11-49 min 90% A and 10% B, 50-59 min: 50% A and 50% B, 60 min: 100% B.

The crude sample of fraction 3 was diluted in MeCN (10 mg/ mL) and filtered through a 0.45 µm PTFE membrane filter prior to injection. 20 µL of sample was injected into an InertSustain C18 column (3 µm, 250 mm x 4.6 mm i,d.) and eluted with mobile phase A (0.1% formic acid) and B (0.1% formic acid in MeCN). The flow rate was 0.5 mL/min. The column temperature was 40°C. A gradient elution at 10 min intervals was performed as follows: 0-10 min: 90% A and 10% B, 11-49 min 90% A and 10% B, 50-59 min: 50% A and 50% B, 60 min: 100% B.

ESI-LC-TOFMS/MS

Liquid chromatography/mass spectra (LCMS) were acquired using a Waters Xevo QTOF mass spectrometer equipped with an Acquty UPLC HPLC system. The heated capillary and spray voltage were maintained at 250°C and 4.5 kV, respectively. Nitrogen was operated at 80 psi for sheath gas flow rate and 20 psi for auxiliary gas flow rate. The full scan mass spectra from *m/z* 50-5000 were acquired in the positive ion mode with a scan speed of one scan per second. The MS/MS collision gas was helium with collision energy of 30% of the 5 V end-cap maximum ticking voltage. The purity of compound **1** was assumed to be about 90% from the eluates of the reverse phase HPLC at the final step of purification. And used directly for bioassays in the present study.

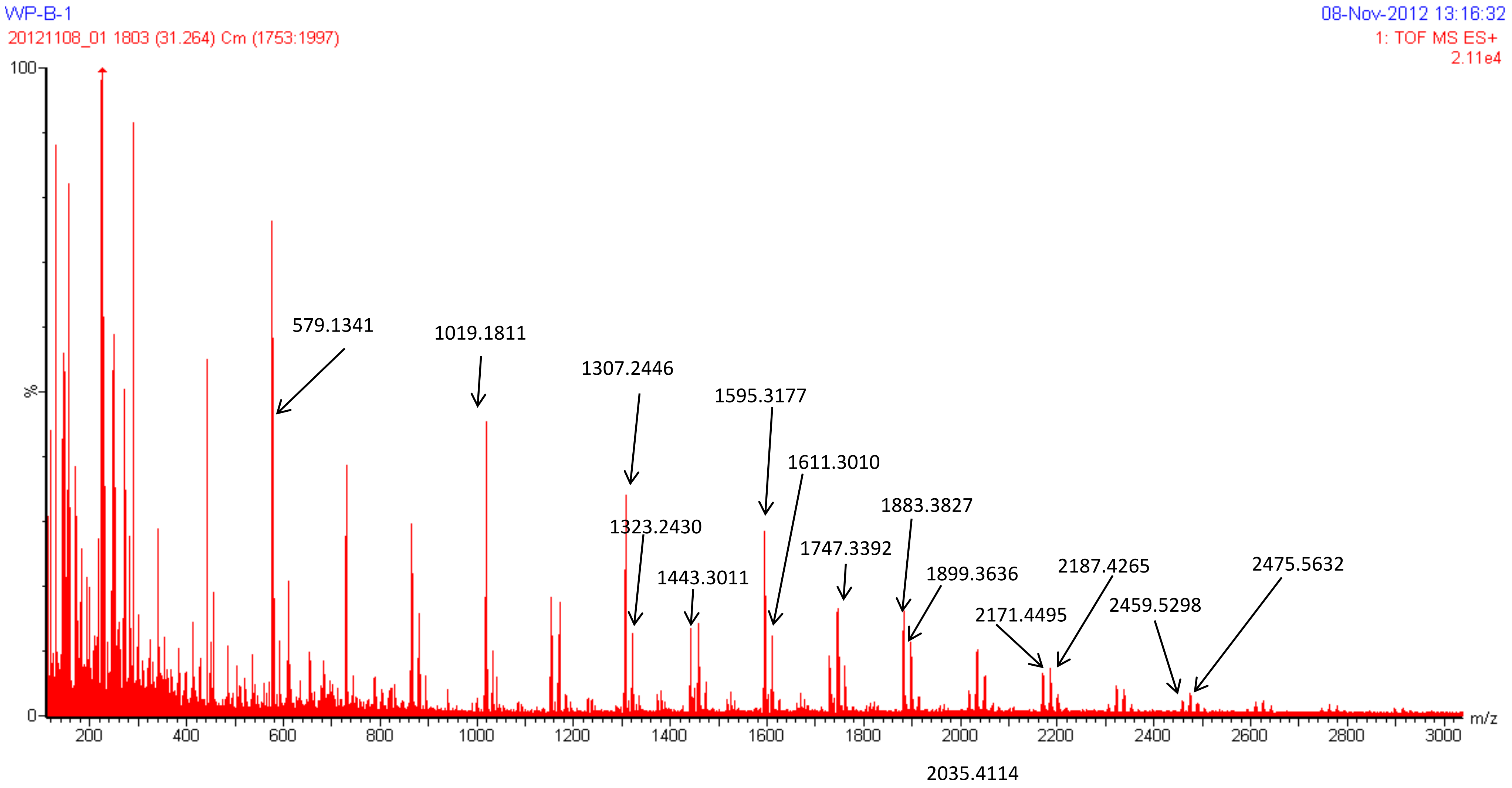

Supplementary Figure 11| ESI-TOF MS spectrum of compound **1**.

30V

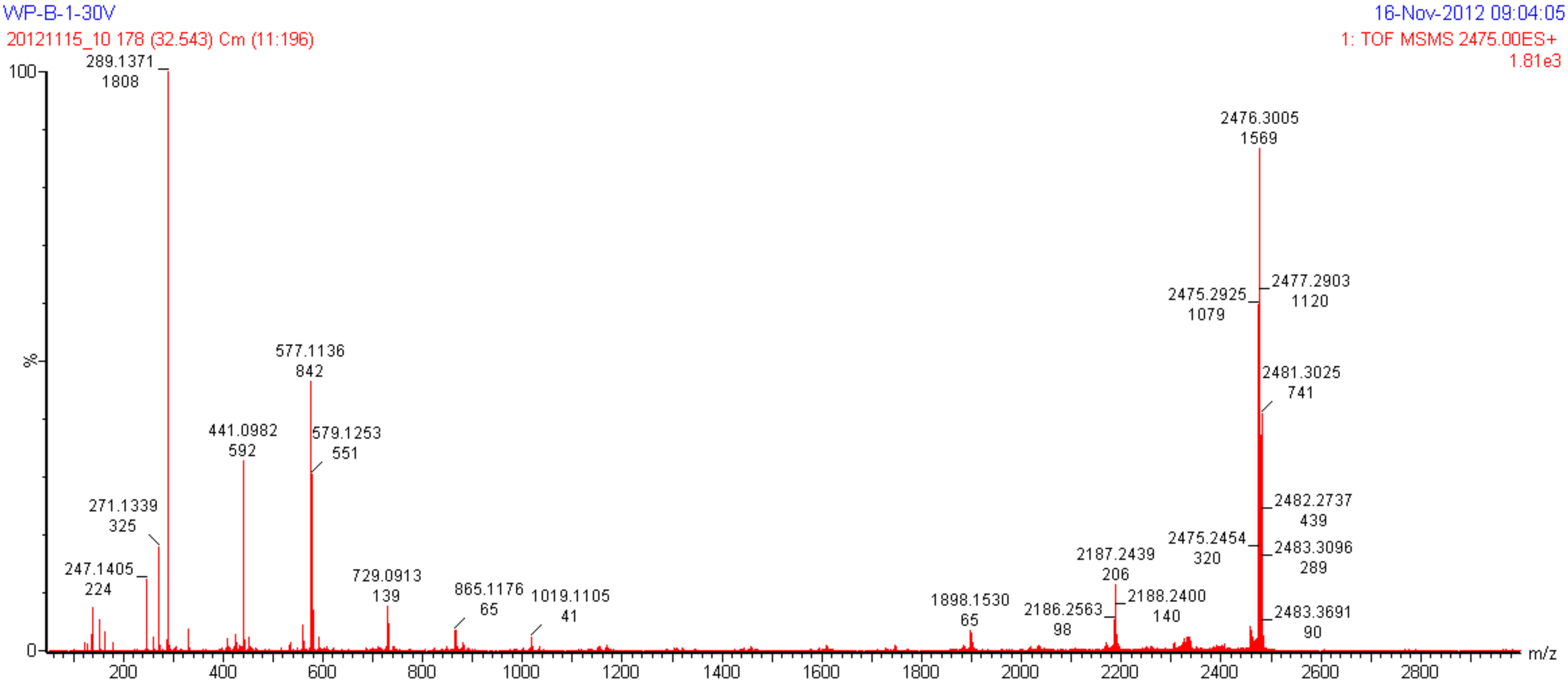

WP-B-1-30V
20121115_10 178 (32.543) Cm (11:196)
16-Nov-2012 09:04:05
1: TOF MSMS 2475.00ES+
1.81e3
100
%
0
289.1371
1808
2476.3005
1569
2477.2903
1120
2475.2925
1079
577.1136
842
2481.3025
741
441.0982
592
579.1253
551
2482.2737
439
271.1339
325
2475.2454
320
2483.3096
289
247.1405
224
2187.2439
206
729.0913
139
2188.2400
140
865.1176
65
1019.1105
41
1898.1530
65
2186.2563
98
2483.3691
90
200
400
600
800
1000
1200
1400
1600
1800
2000
2200
2400
2600
2800
m/z

40V

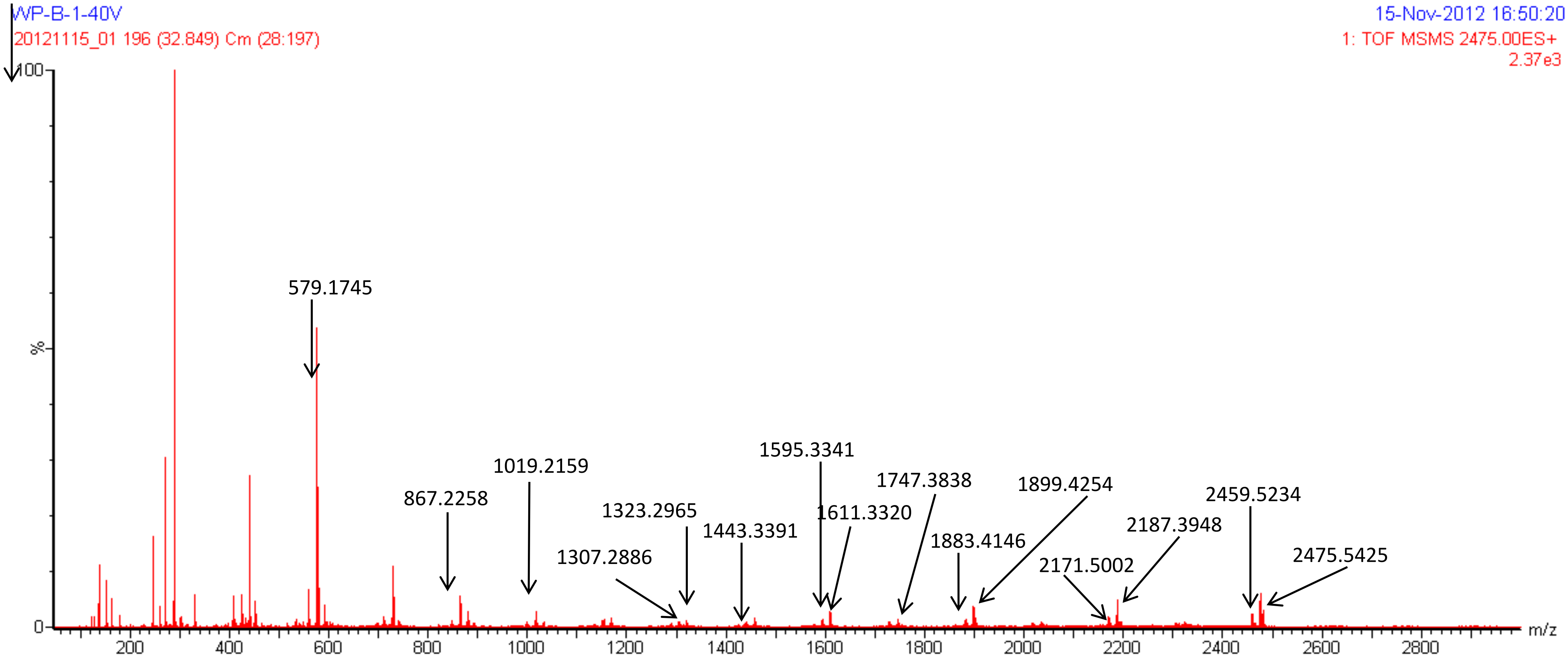
WP-B-1-40V
20121115_01 196 (32.849) Cm (28:197)
15-Nov-2012 16:50:20
1: TOF MSMS 2475.00ES+
2.37e3
100
%
0
579.1745
867.2258
1019.2159
1307.2886
1323.2965
1443.3391
1595.3341
1611.3320
1747.3838
1883.4146
1899.4254
2171.5002
2187.3948
2459.5234
2475.5425
200
400
600
800
1000
1200
1400
1600
1800
2000
2200
2400
2600
2800
m/z

50V

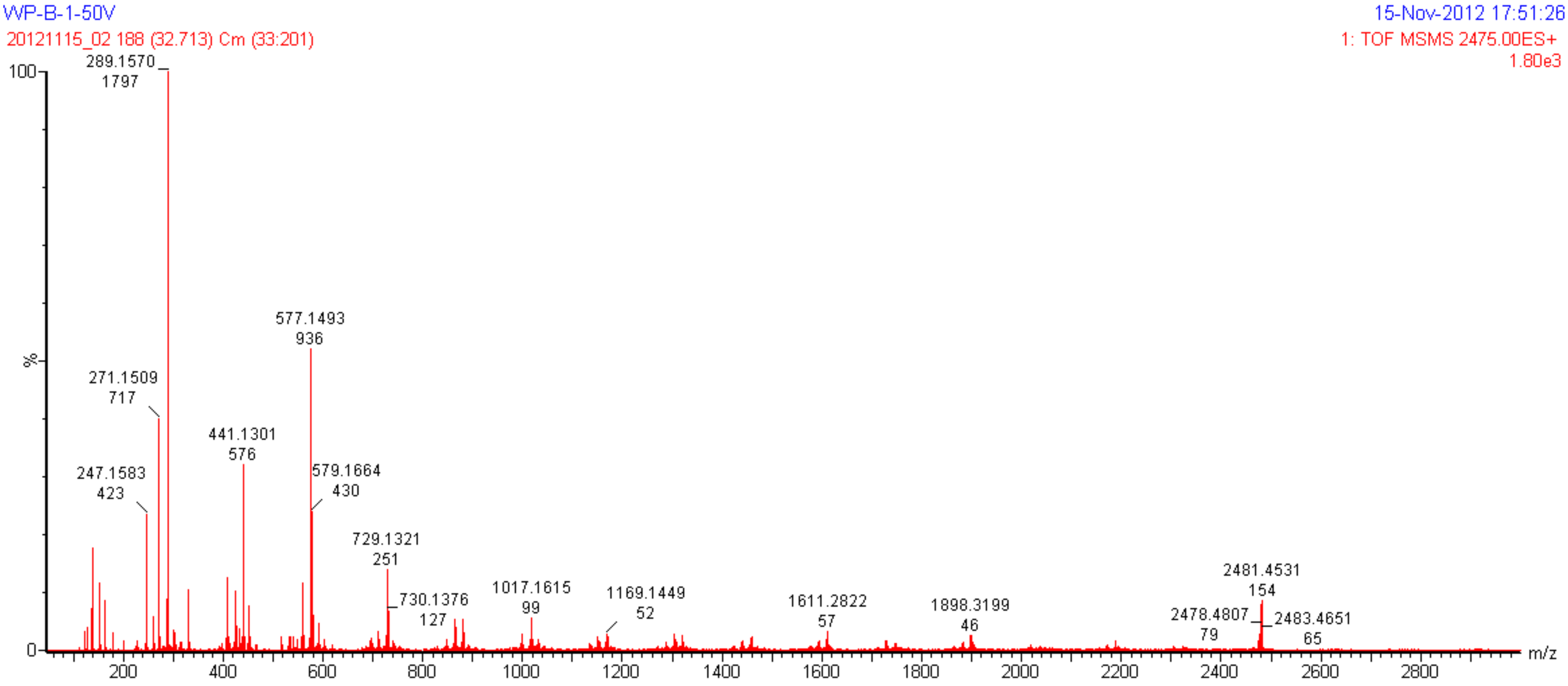
WP-B-1-50V
15-Nov-2012 17:51:26
20121115_02 188 (32.713) Cm (33:201)
1: TOF MSMS 2475.00ES+
1.80e3
100
%
0
289.1570
1797
577.1493
936
271.1509
717
441.1301
576
247.1583
423
579.1664
430
729.1321
251
730.1376
127
1017.1615
99
1169.1449
52
1611.2822
57
1898.3199
46
2481.4531
154
2478.4807
79
2483.4651
65
200
400
600
800
1000
1200
1400
1600
1800
2000
2200
2400
2600
2800
m/z

60V

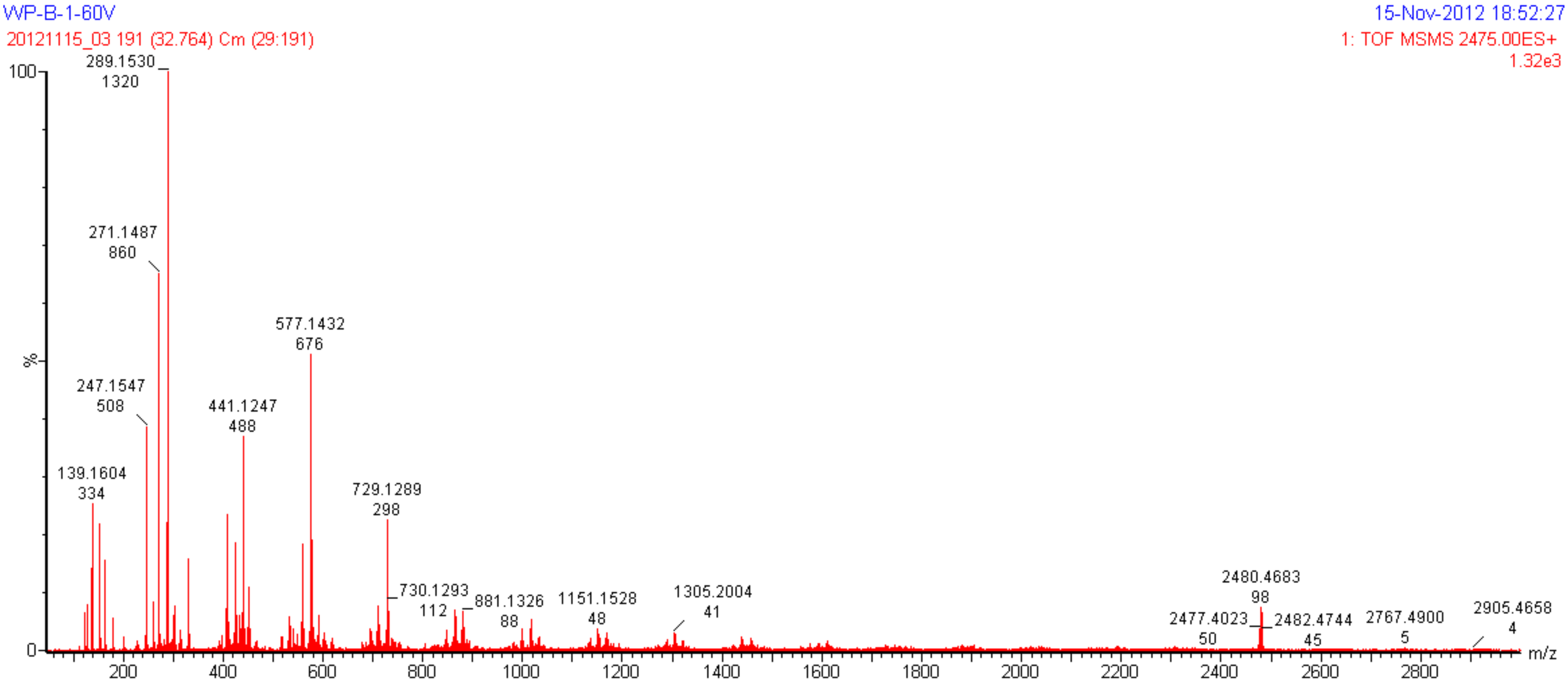

Supplementary Figure 12 | ESI-MS/MS spectra of compound **1** with 30-60V.

Supplementary Figure 13 | Full images of Western blot analyses of the expression of the cancer-promoting gene *FABP5* by treatment of test compounds.

The experimental procedures are described in Biochemical methods and Fig.7.

(a) Full-length membrane in visible light. This experiment was done duplicate (1 and 2). (b) Full-length membrane of Figure 7b (indicated by broken line). Protein (FABP5 or GAPDH) was visualized by chemiluminescent.

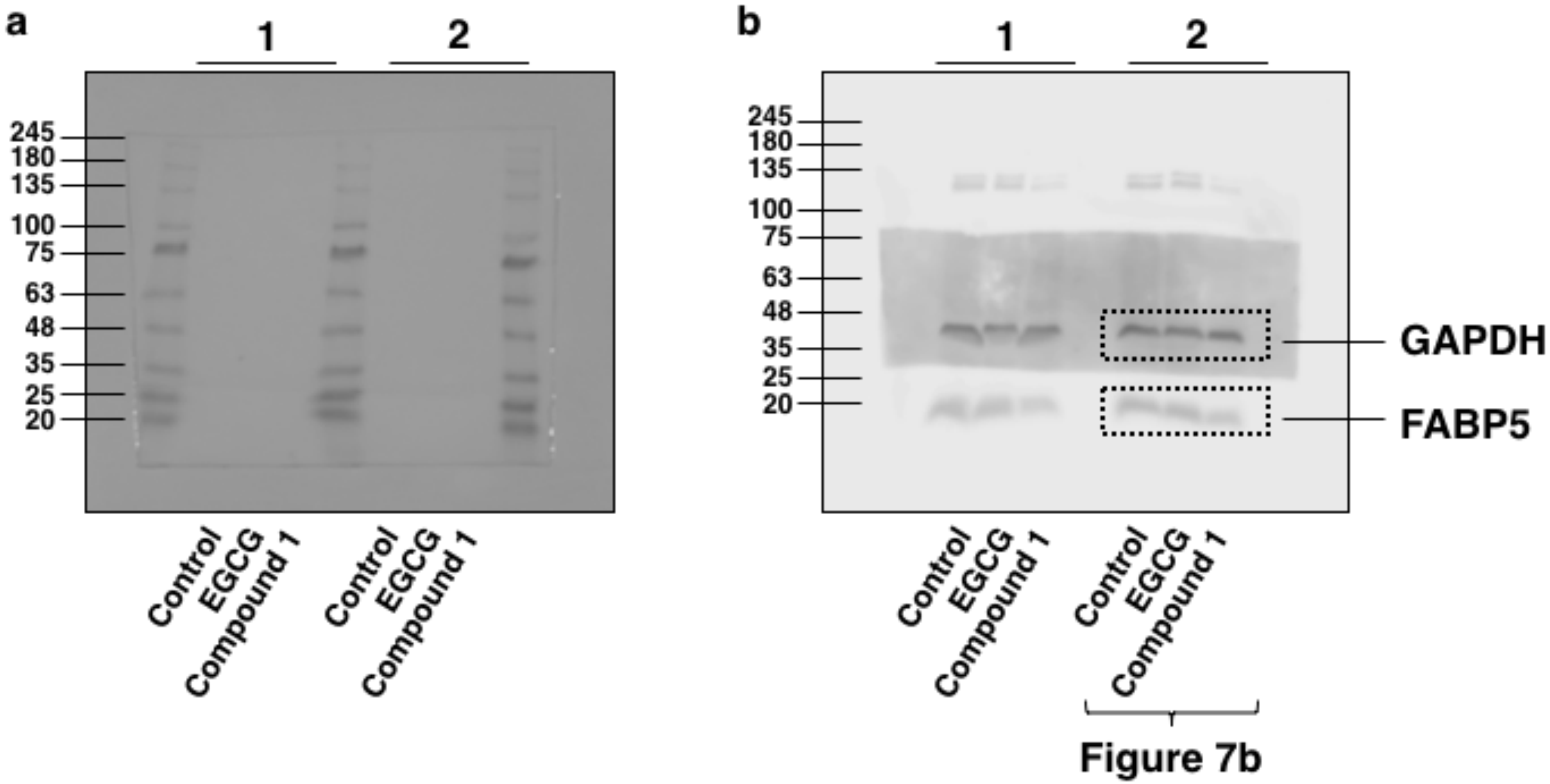

Biochemical Methods

Cell lines, cell culture and reagents.

Human PC-3 prostate cancer cell lines were purchased from the Health Science Research Resources Bank. The cells were maintained in monolayer culture at 37 °C under 5% CO_2 in RPMI-1640 (SIGMA, R8755) supplemented with 10% charcoal-stripped fetal bovine serum (Biological Industries, No. 04-201-1) and 1% antibiotic-antimycotic mixed stock solution (Nacalai Tesque, No. 09366-44). The procedure for preparation of test compounds was described in the following section.

Preparation of test compounds.

Preparation of samples for bioassay was carried out as described in the previous study[1]. The test compounds, various procyanidins, were dissolved in 60% (v/v) ethanol and prepared for the concentrated solution (10-fold concentration) with the midium (RPMI 1640, Hyclone, Thermo Scientific) containing 10% charcoal-stripped FBS (Hyclone, Thermo Scientific) and antibiotic/antimycotic solution (Nacalai Tesque). The concentrated solution was filtrated by using a 0.2-µm filter (Sartorius Stedim Biotech, Goettingen, Germany). In the experiments, the concentrated solution was added to the samples to a final concentration as indicated. Final concentration of ethanol of each sample including the control is 6% v/v. The medium used during treatment periods is the same as the growth medium (RPMI 1640, Hyclone, Thermo Scientific) supplemented with charcoal-stripped 10% FBS (Hyclone, Thermo Scientific) and antibiotic/antimycotic solution (Nacalai Tesque).

Cell count.

Experimental procedure for cell count was carried out as described in the previous study[3]. Cells were plated in dishes and allowed to reach 50% confluent growth. The cells were treated with the indicated concentrations of EGCG or compound 1 for 48 h. The cells treated with the above test compounds were trypsinized. After adding the culture medium to

each well, they were agitated by pipetting. The number of cells was measured with the hemocytometer. After treatment of PC-3 cells with test compound such as EGCG or compound 1 for 48 h, the cells were observed under the microscope and the number of cells was counted.

qPCR (quantitative real-time PCR).

Experimental procedure for bioassay by qPCR was carried out as described in the previous study[1]. Cells were plated in 6-well plates and allowed to reach 50% confluent growth. The cells were treated with the indicated concentrations of test compounds such as compound **1** (30 μmol/L) for 48 h. Total RNA of these cells was extracted using the Plant RNA Purification Reagent (Invitrogen No. 12322-012), and 1 μg of total RNA was reverse transcribed into cDNA using the ReverTra Ace qPCR RT Master Mix (Toyobo No. FSQ-301). qPCR analyses were performed with the StepOne Real-Time PCR system (Applied Biosystems) using THUNDERBIRD® SYBR® qPCR Mix (Toyobo No. QPS-201). The sequences of the FABP5, GAPDH and 18S rRNA RPL27 primers for qPCR are as follows[1]:

FABP5 (Forward): 5'-GCTGATGGCAGAAAAACTCAGA-3', (Reverse): 5'-CCTGATGCTGAACCAATGCA-3'

GAPDH (Forward): 5'-CAGCCTCAAGATCATCAGCA-3', (Reverse): 5'-GGTGCTAAGCAGTTGGTGGT-3'

18S rRNA RPL27 (Forward):5'-CGGCTACCACATCCAAGGAA-3', (Reverse): 5'-GCTGGAATTACCGCGGCT-3'

Western blot analysis.

Experimental procedure for western blot analysis was carried out as described in the previous study[1]. Cells were plated in 60 mm cell culture dishes and allowed to reach 50% confluent growth. The cells were treated with the indicated concentrations of test compounds such as compound **1** (30 μmol/L) for 48 h. These cells were lysed for protein extraction using Lysis buffer with protease inhibitor cocktail (Nacalai Tesque No. 25955-11). After protein concentrations were determined by the Bradford assay, 50 μg of protein per sample was fractionated by SDS/PAGE. Western blot analysis was carried out using the appropriate antibodies and chemiluminescent substrates (Thermo Scientific # NCI3106). Signals were detected with the Image Quant LAS4000 Mini (GE Healthcare Biosciences)[1]. The antibodies were used as follows. FABP5 (D1A7T) Rabbit mAb #39926 (Cell Signaling Technology). GAPDH antibody (FL-335): sc-25778 (Santa Cruz Biotechnology)[1].

Measurement of apoptosis by assay for caspase-3 activity.

Measurement of apoptosis-inducible activity was carried out as described in the previous study[1].Assays for caspase-3 activity were carried out using BD Cytofix/Cytoperm™ Kit (BD Biosciences, No. 554714), according to the manufacture's protocol. Purified rabbit anti-active caspase-3 (BD Pharmingen™, No. 559565) was used as the first antibody (1st Ab) and FITC-conjugate anti-rabbit Ig G (Jackson ImmunoResearch, No. 711-096-152) was used for the second antibody (2nd Ab). Briefly, after treatment of cells with 30 μmol/L of EGCG, 30 μmol/L of compound **1** or 500 nmol/L of carnitine palmitoyltransferase (CPT) for 48 h, the cells were collected and prepared by the same method as described in cell cycle analysis. The cells were diluted in PBS and fixed with BD Cytofix/Cytoperm™ Fixation and Permilization Solution on ice in the dark for 20 min. The cells were washed with the washing buffer and then reacted with 1st Ab at room temperature. Next, the cells were washed with the washing buffer and then reacted with 2nd Ab at room temperature. After the reaction, the cells were diluted in PBS, and flow cytometry was performed with FACScan (Becton Dickinson, Japan), and the data obtained were analyzed utilizing Cell Quest Software. For each sample, 1 x 10^4 cells were recorded.

Cell cycle analysis.

Cell cycle analysis was carried out as described in the previous study[1]. Cells were plated in 90 mm cell culture dishes and grew to reach 50% confluent. Effects of test compounds (EGCG or compound **1**) on the phase distribution of cell cycle were assessed by flow cytometry. After treatment of cells with 30 μmol/L of EGCG or compound **1** for 48 h, floated cells were discarded by aspiration and the attached cells were trypsinized (Nacalai Tesque, No. 35556-44) and thereafter washed twice with cold PBS, and centrifuged. The pellets were used for sample preparation for flow cytometry using BD Cycletest™ Plus DNA Reagent Kit (BD Biosciences, No.340242), according to the manufacturer's protocol. Flow cytometry was performed with a FACSCalibur flow cytometer (BD Biosciences), and the data were analyzed utilizing Cell Quest software. For each sample, 1×10^4 cells were recorded.

Invasion assay

Invasion assay was carried out as described in the previous study[1]. The *in vitro* invasion assay was performed using BioCoat Matrigel invasion chambers (24-well plate, 8 μm pore size; BD Biosciences No. 354480). PC-3 cells were seeded on the upper parts of the Transwell chamber at 1×10^5 cells in 500 μL serum-free medium with 30 μmol/L EGCG or compound **1**. The chamber was placed into the 24-well plate, which contained 750 μL of RPMI1640 containing 10% charcoal-stripped FBS. These cultures were incubated at 37 °C in a CO_2 incubator for 36 h. The cells that invaded to the lower surface of the filter were fixed and stained using Diff-Quick Stain Kit (Symex, Kobe, Japan). The invasive cells were counted in three random fields per chamber.

Statistical analysis.

Each experiment was performed at least three times. Data were expressed as the means ± standard deviation (S.D.). Statistical significance of differences between groups was analysed by Student's t-tests or one-way analysis of variance (ANOVA) followed by Dunnett's post hoc test for multiple comparison. All statistical analyses were performed using GraphPad Prism 7 (GraphPad Software, San Diego, CA). $^*P < 0.05$ or $^{**}P<0.01$ was considered statistically significant.

Supplementary references

1. Takanashi, K., Suda, M., Matsumoto, K., Ishihara, C., Toda, K., Kawaguchi, K., Senga, S., Kobayashi, N., Ichikawa, M., Katoh, M., Hattori, Y., Kawahara, S–i., Umezawa, K., Fujii, H., & Makabe, H. Epicatechin oligomers longer than trimers have anti-cancer activities, but not the catechin counterparts. *Sci. Rep.*, **7**, 7791 (2017).

2. Hamauzu, Y., Kishida, H., & Yamazaki, N. Gastroprotective property of *Pseudocydonia sinensis* fruit jelly on the ethanol-induced gastric lesions in rats. *J. Funct. Foods* **48**, 275-282 (2018).

3. Fujii, W., Toda, K., Kawaguchi, K., Kawahara, S.-i., Katoh, M., Hattori, Y., Fujii, H. &_Makabe, H. Syntheses of prodelphinidin B3 and C2, and their antitumor activities through cell cycle arrest and caspase-3 activation. *Tetrahedron* **69**, 3543 (2013).
